# Lactic Acid Bacteria in Finfish—An Update

**DOI:** 10.3389/fmicb.2018.01818

**Published:** 2018-08-10

**Authors:** Einar Ringø, Seyed Hossein Hoseinifar, Koushik Ghosh, Hien Van Doan, Bo Ram Beck, Seong Kyu Song

**Affiliations:** ^1^Faculty of Bioscience, Fisheries and Economics, Norwegian College of Fishery Science, UiT The Arctic University of Norway, Tromsø, Norway; ^2^Department of Fisheries, Faculty of Fisheries and Environmental Sciences, Gorgan University of Agricultural Sciences and Natural Resources, Gorgan, Iran; ^3^Aquaculture Laboratory, Department of Zoology, The University of Burdwan, Bardhaman, India; ^4^Department of Animal and Aquatic Sciences, Faculty of Agriculture, Chiang Mai University, Chiang Mai, Thailand; ^5^School of Life Science, Handong University, Pohang, South Korea

**Keywords:** lactic acid bacteria (LAB), finfish, probiotics, probiotic bacteria, fish immunity, aquaculture

## Abstract

A complex and dynamic community of microorganisms, play important roles within the fish gastrointestinal (GI) tract. Of the bacteria colonizing the GI tract, are lactic acid bacteria (LAB) generally considered as favorable microorganism due to their abilities to stimulating host GI development, digestive function, mucosal tolerance, stimulating immune response, and improved disease resistance. In early finfish studies, were culture-dependent methods used to enumerate bacterial population levels within the GI tract. However, due to limitations by using culture methods, culture-independent techniques have been used during the last decade. These investigations have revealed the presence of *Lactobacillus, Lactococcus, Leuconostoc, Enterococcus, Streptococcus, Carnobacterium, Weissella*, and *Pediococcus* as indigenous species. Numerous strains of LAB isolated from finfish are able to produce antibacterial substances toward different potential fish pathogenic bacteria as well as human pathogens. LAB are revealed be the most promising bacterial genera as probiotic in aquaculture. During the decade numerous investigations are performed on evaluation of probiotic properties of different genus and species of LAB. Except limited contradictory reports, most of administered strains displayed beneficial effects on both, growth—and reproductive performance, immune responses and disease resistance of finfish. This eventually led to industrial scale up and introduction LAB-based commercial probiotics. Pathogenic LAB belonging to the genera *Streptococcus, Enterococcus, Lactobacillus, Carnobacterium*, and *Lactococcus* have been detected from ascites, kidney, liver, heart, and spleen of several finfish species. These pathogenic bacteria will be addressed in present review which includes their impacts on finfish aquaculture, possible routes for treatment. Finfish share many common structures and functions of the immune system with warm-blooded animals, although apparent differences exist. This similarity in the immune system may result in many shared LAB effects between finfish and land animals. LAB-fed fish show an increase in innate immune activities leading to disease resistances: neutrophil activity, lysozyme secretion, phagocytosis, and production of pro-inflammatory cytokines (*IL-1*β*, IL-6, IL-8*, and *TNF-*α). However, some LAB strains preferentially induces *IL-10* instead, a potent anti-inflammatory cytokine. These results indicate that LAB may vary in their immunological effects depending on the species and hosts. So far, the immunological studies using LAB have been focused on their effects on innate immunity. However, these studies need to be further extended by investigating their involvement in the modulation of adaptive immunity. The present review paper focuses on recent findings in the field of isolation and detection of LAB, their administration as probiotic in aquaculture and their interaction with fish immune responses. Furthermore, the mode of action of probiotics on finfish are discussed.

## Introduction

Optimal gastrointestinal (GI) functionality is essential for sustainable animal production. Effective functionality of the finfish GI tract and its gut microbiota play and important role in host health (Ringø et al., 2003; Round and Mazmanian, 2009), and several complex mechanisms are involved, and in the absence of gut microbiota, normal immune development, and function are impaired. Therefore it is crucial to increase our knowledge on beneficial gut bacteria, for example lactic acid bacteria (LAB) colonizing the GI tract, in the context of improved growth performance and health.

LAB are classified in phylum Firmicutes, class Bacilli, and order Latobacillales. They are Gram-positive, non-endosporing, with rod-shaped or coccid morphology, are catalase- and oxidase-negative and most of them are non-motile. The growth optimum of LAB is generally at pH 5.5–5.8, and they have complex nutritional requirements. They are divided into homofermentative and heterofermentative; homofermentative produce lactic acid from sugars, while heterofermentative produce lactic acid, acetic acid or alcohol, and carbon dioxide. A favorable trait of LAB is; they produce growth inhibition substances such as bacteriocins, hydrogen peroxide, diacyls, etc.; prevent proliferation of pathogenic—and spoilage bacteria in food (Alakomi et al., 2000; De Vuyst and Leroy, 2007), as well as adherence and colonization of pathogens in the digestive tract (Li et al., 2018).

LAB genera include rods; *Carnobacterium, Dolosigranulum, and Lactobacillus, cocci; Aerococcus, Alloiococcus, Enterococcus, Lactococcus, Leuconostoc, Oenococcus, Pediococcus, Streptococcus, Tetragenococcus*, and *Vagococcus*, and the coccoid or rod-shaped genus Weissella (Walter, 2008; Ventura et al., 2009; Fusco et al., 2015). They are isolated from different sources; e.g., plant material, fruits, dairy products, fermented meat, cavities of humans as well as the gastrointestinal (GI) tract of finfish (e.g., Ventura et al., 2009; Merrifield et al., 2014; Ringø et al., 2016).

The fish gut microbiota plays an important role in GI tract development, digestive function, mucosal tolerance, stimulating the host immune response, and protection against infections (e.g., Rawls et al., 2004, 2006; Gómez and Balcázar, 2008; German, 2009; Ray et al., 2012; Maiuta et al., 2013; Piazzon et al., 2017; Tarnecki et al., 2017; Li et al., 2018; Wang et al., 2018). Furthermore, host-microbe interactions are influenced by complex host genetics and environment. In a recent review, Lescak and Milligan (2017) suggested teleost as model organisms to understand host-microbe interactions, as traditional mammalian studies can be limited by isogenic strains, small sample sizes, limited statistical power and indirect characterization of gut microbiota from fecal samples.

As the GI tract in fish is one of the most important interfaces with the environment exposed to potential pathogens, it is of importance to evaluate the presence of beneficial bacteria such as LAB in the GI tract, as autochthonous bacteria rapidly colonize the digestive tract at early developmental larval stages of finfish (Ringø et al., 1996).

During the last 20 years, an impressive amount of knowledge has been published on LAB in finfish intestine, their potential as probiotics, pathogenicity and their effect on the immune system (Ringø and Gatesoupe, 1998; Ringø, 2004; Ringø et al., 2005, 2012a,b; Gatesoupe, 2008; Lauzon and Ringø, 2011; Merrifield et al., 2014; Ringø and Song, 2016; Zhou Z. et al., 2018). To avoid duplication, studies reviewed in the aforementioned reviews are not addressed in the present paper. The current review aimed to present an updated overview of recently published data on LAB, and on LAB data not mention in the aforementioned reviews on the topics; on LAB in the GI tract of finfish, antagonistic ability, health benefits as probiotics, pathogenicity, and on immunostimulation.

## Lactic acid bacteria (LAB) in the gastrointestinal (GI) tract

The GI tract microbiota in endothermic animals as well as fish is divided into; the GI lumen microbiota (the allochthonous), and those that adhere to the mucosal surface (the autochthonous microbiota). In most studied showed in Table 1 have, however, characterized the allochthonous gut microbiota.

**Table 1:** Lactic acid bacteria (LAB) in the gastrointestinal tract of finfish.

**LAB species isolated**	**Isolated from**	**“*Segments*” of the GI tract**	**References**
LAB^*^	Tasmanian Atlantic salmon (*Salmo salar*)	Fecal content	Neuman et al., 2015
	Persian sturgeon (*Acipenser persicus*)	EI; auto and allo	Ovissipour et al., 2014
	Beluga (*Huso huso*)	EI; allo	Adel et al., 2017
	Oscar (*Astronotus ocellatus*)	EI; auto	Hoseinifar et al., 2016a
	Tilapia (*Oreochromis niloticus*)	EI; allo	Standen et al., 2016
	Nile tilapia (*Oreochromis niloticus*)	EI; content	Boonanuntanasarn et al., 2017
*Carnobacterium*	Rainbow trout (*Oncorhynchus mykiss*)	DI; auto and allo	Lyons et al., 2017a
	Rainbow trout	DI; auto and allo	Huyben et al., 2017
	Rainbow trout	EI; auto	Bruni et al., 2018
	Atlantic salmon (*Salmo salar*)	Fecal content	Zarkasi et al., 2016
	Atlantic salmon	DI; content	Gajardo et al., 2017
	Atlantic salmon	EI; content	Rudi et al., 2018
	Turbot (*Scophthalmus maximus*)	EI; auto	Yang et al., 2018
	Fine flounder (*Paralichthys adspersus*)	EI: content	Salas-Leiva et al., 2017
	Northern snakehead (*Channa argus*)	EI: content	Miao et al., 2018
*C. divergens*	Rainbow trout	EI; allo	Bruni et al., 2018
*Lactobacillus*	Rainbow trout	DI; auto and allo	Lyons et al., 2016
	Rainbow trout	PI; auto and allo	Bahramian and Parsa, 2017
	Rainbow trout	DI; auto and allo	Lyons et al., 2017a
	Rainbow trout	DI; auto and allo	Lyons et al., 2017b
	Rainbow trout	DI; auto and allo	Huyben et al., 2017
	Atlantic salmon	Fecal content	Zarkasi et al., 2016
	Atlantic salmon	EI; Digesta samples	Dehler et al., 2017a
	Atlantic salmon	DI; Digesta samples	Dehler et al., 2017b
	Atlantic salmon	DI; allo	Zarkasi et al., 2016
	Atlantic salmon	DI; content	Gajardo et al., 2017
	Atlantic salmon	PI and DI; auto	Lavoie et al., 2018
	Atlantic salmon	EI; auto and allo	Rimoldi et al., 2018
	Arctic charr (*Salvelinus alpinus*)	PI; auto and allo DI; auto and allo	Nyman et al., 2017
	Regal peacock (*Aulonocara stuartgranti*)	EI; allo	Mirzapour-Rezaee et al., 2017
	Largemouth bass (*Micropterus salmoides*)	EI; content	Zhou M. et al., 2018
	European sea bass (*Dicentrarchus labrax*)	EI; content	Torrecillas et al., 2017
	Fine flounder	EI: content	Salas-Leiva et al., 2017
	Gibel carp (*Carassius auratus gibelio*)	EI: content	Wu et al., 2018
	Loach (*Paramisgurnus dabryanus*)	EI: auto and allo	Gao et al., 2017
	Zebrafish (*Danio rerio*)	EI: content	Yang et al., 2017
*Lb. aviarius*	Tilapia	EI; auto and allo	Standen et al., 2015
*Lb. aviaries* subsp. *arafinosus*	White sea bream (*Diplodus sargus*)	EI; auto and allo	Guerreiro et al., 2018a
*Lb. brevis*	Tilapia	EI; content	Del‘Duca et al., 2015
*Lb. crispatus*/ *Lb. amylovorus*	Gilthead sea bream (*Sparus aurata*)	EI; auto and allo	Serra et al., 2018
*Lb. crispatus*	White sea bream	EI; auto and allo	Guerreiro et al., 2018a
	European sea bass	EI; auto and allo	Guerreiro et al., 2018b
*Lb. collinoides*	Tilapia	EI; content	Del‘Duca et al., 2015
*Lb. coryniformis*	Tilapia	EI; content	Del‘Duca et al., 2015
*Lb. farciminis*	Tilapia	EI; content	Del‘Duca et al., 2015
*Lb. gallinarum*	White sea bream	EI; auto and allo	Guerreiro et al., 2018a
*Lb. johnsonii*	European sea bass	EI; content	Torrecillas et al., 2017
*Lb. paracasei* subsp*. paracasei*	Rainbow trout	EI; content	Popovic et al., 2017
*Lb. reuteri*	Rainbow trout	EI; content	Huyben et al., 2018
*Lb. sakei*	Rainbow trout	DI: auto and allo	Didinen et al., 2018
*Lactococcus*	Rainbow trout	DI; auto and allo	Lyons et al., 2016
	Rainbow trout	DI; auto and allo	Lyons et al., 2017a
	Rainbow trout	DI; auto and allo	Lyons et al., 2017b
	Rainbow trout	DI; auto and allo	Huyben et al., 2017
	Atlantic salmon	Fecal content	Zarkasi et al., 2016
	Atlantic salmon	Digesta samples	Dehler et al., 2017b
	Atlantic salmon	DI; content	Gajardo et al., 2017
	Atlantic salmon	EI; content	Rudi et al., 2018
	Atlantic salmon	EI; auto and allo	Rimoldi et al., 2018
	Arctic charr	PI; auto and allo DI; auto and allo	Nyman et al., 2017
	Grass carp (*Ctenopharyngodon idella*)	NI	Tran et al., 2017
	Gibel carp	EI: content	Wu et al., 2018
	Northern snakehead	EI: content	Miao et al., 2018
	Loach	EI: auto and allo	Gao et al., 2017
	Zebrafish	EI: content	Yang et al., 2017
	Zebrafish	EI: content	Zhou L. et al., 2018
*L. garvieae*	Pirarucu (*Arapaima gigas*)	EI; auto and allo	do Vale Pereira et al., 2017
	Turbot	EI; auto	Yang et al., 2018
*L. lactis*	Grass carp	EI; auto and allo	Dong et al., 2017
*L. garvieae*	Rainbow trout	DI: allo	Didinen et al., 2018
*L. lactis* subsp. *cremoris*	Rainbow trout	DI: allo	Didinen et al., 2018
*L. lactis* subsp*. lactis*	Pirarucu	EI; auto and allo	do Vale Pereira et al., 2017
*L. piscium*	European sea bass	EI; content	Torrecillas et al., 2017
*L. raffinolactis*	Grass carp	EI; auto	Li et al., 2015
	Grass carp	EI; auto and allo	Dong et al., 2017
Leuconostocaceae	Rainbow trout	EI; auto and allo	Huyben et al., 2018
*Leuconostoc*	Rainbow trout	DI; auto and allo	Lyons et al., 2016
	Rainbow trout	DI; auto and allo	Lyons et al., 2017b
	Rainbow trout	DI; auto and allo	Huyben et al., 2017
	Atlantic salmon	Digesta samples	Dehler et al., 2017b
	Atlantic salmon	DI; content	Gajardo et al., 2017
	Atlantic salmon	EI; auto and allo	Rimoldi et al., 2018
	Arctic charr	PI; auto and allo DI; auto and allo	Nyman et al., 2017
	Tilapia	EI; auto and allo	Standen et al., 2015
	Loach	EI: auto and allo	Gao et al., 2017
*Pediococcus*	Atlantic salmon	DI; content	Gajardo et al., 2017
	Atlantic salmon	PI and DI; auto	Lavoie et al., 2018
	Turbot	EI; auto	Yang et al., 2018
*P. acidilactici*	Rainbow trout	DI: allo	Didinen et al., 2018
Streptococcacceae	Rainbow trout	EI; auto and allo	Huyben et al., 2018
	Atlantic salmon	PI and DI; auto	Lavoie et al., 2018
*Streptococcus*	Rainbow trout	DI; auto and allo	Lyons et al., 2016
	Rainbow trout	DI; auto and allo	Lyons et al., 2017a
	Rainbow trout	DI; auto and allo	Lyons et al., 2017b
	Atlantic salmon	Fecal content	Zarkasi et al., 2016
	Atlantic salmon	Digesta samples	Dehler et al., 2017a
	Atlantic salmon	Digesta samples	Dehler et al., 2017b
	Atlantic salmon	EI; auto and allo	Rimoldi et al., 2018
	European sea bass	EI; content	Torrecillas et al., 2017
	Turbot	EI; auto	Yang et al., 2018
	Fine flounder	EI: content	Salas-Leiva et al., 2017
	Pirarucu	EI; auto and allo	do Vale Pereira et al., 2017
	Northern snakehead	EI: content	Miao et al., 2018
*S. luteciae*	Rainbow trout	DI; auto and allo	Huyben et al., 2017
	Arctic charr	PI; auto and allo DI; auto and allo	Nyman et al., 2017
*S. sobrinus*	Rainbow trout	DI; auto and allo	Huyben et al., 2017
	Arctic charr	PI; auto and allo DI; auto and allo	Nyman et al., 2017
*Enterococcus*	Rainbow trout	DI; auto and allo	Lyons et al., 2016
	Rainbow trout	DI; auto and allo	Lyons et al., 2017a
	Atlantic salmon	EI; auto and allo	Rimoldi et al., 2018
	Turbot	EI; auto	Yang et al., 2018
	Zebrafish	EI: content	Yang et al., 2017
	Zebrafish	EI: content	Zhou L. et al., 2018
*E. faecalis*	Mrigal (*Cirrhinus mrigala*)	EI; allo	Shahid et al., 2017
*E. faecium*	European sea bass	EI; content	Torrecillas et al., 2017
	Tilapia	EI; auto and allo	Standen et al., 2015
	Pirarucu	EI; auto and allo	do Vale Pereira et al., 2017
*Vagococcus*	Rainbow trout	DI; auto and allo	Lyons et al., 2017a
	Atlantic salmon	DI; content	Gajardo et al., 2017
	Atlantic salmon	EI; content	Rudi et al., 2018
	Fine flounder	EI: content	Salas-Leiva et al., 2017
*Weissella*	Rainbow trout	DI; auto and allo	Lyons et al., 2016
	Rainbow trout	DI; auto and allo	Lyons et al., 2017a
	Rainbow trout	DI; auto and allo	Lyons et al., 2017b
	Atlantic salmon	Digesta samples	Dehler et al., 2017b
	Atlantic salmon	DI; content	Gajardo et al., 2017
	Atlantic salmon	EI; content	Rudi et al., 2018
	Atlantic salmon	EI; auto and allo	Rimoldi et al., 2018
	Rohu (*Labeo rohita*)	EI; allo	Shahid et al., 2017
	Tilapia	EI; auto and allo	Standen et al., 2015
	Common snook (*Centropomus undecimalis*)—larvae	Whole larvae	Tarnecki and Rhody, 2017
	Fine flounder	EI: content	Salas-Leiva et al., 2017
*W. paramesenteroides*	Pirarucu	EI; auto and allo	do Vale Pereira et al., 2017
*Bifidobacterium*	Nile tilapia	EI: content	Boonanuntanasarn et al., 2017

* *A no further information was given; EI, entire intestine without pyloric caeca; PI, posterior intestine; DI, distal intestine; auto, autochthonous; allo, allochthonous; NI, no information*.

During the last decades, numerous investigations on the isolations of LAB in finfish have been carried out. According to Merrifield et al. (2014) members belonging to *Lactobacillus, Lactococcus, Leuconostoc, Enterococcus, Streptococcus, Carnobacterium, Pediococcus*, and *Weissella* genera are indigenous species in finfish. In this subsection, results of some investigations published the last 3 years are presented. Readers with special interest in studies not described in the text are recommended to have a closer look at the original papers.

### LAB

In numerous studies, counts of presumptive LAB has been revealed, but without going into further identification. In their study of Persian sturgeon (*Acipenser persicus* L.) larvae fed tuna viscera protein hydrolysate, Ovissipour et al. (2014) reported that culturable LAB counts in the intestinal contents was significantly (*P* < 0.05) higher when the larvae were fed fish protein hydrolysate at the highest inclusion level, 347g kg^−1^, compared to control fed larvae. However, the log LAB counts were only ~3.0 compared to log levels of total counts; ~5.0. In their comprehensive review devoted to dietary effect on gut microbiota of finfish, Ringø et al. (2016) revealed an overview on gut microbiota due to seasonal variations. It is also worth mentioning that seasonal variations of *Lactobacillus* and putative pathogenic bacteria density occurs in aquaculture system (Resende et al., 2015). Neuman et al. (2015) evaluated the effect of diets, smolt-, summer, and growing diets, on fecal microbiota of farmed Tasmanian Atlantic salmon (*Salmo salar* L.) and revealed a decrease in LAB numbers during rearing from November to May. Furthermore, Hoseinifar et al. (2016a) revealed that increasing supplementation of xylooligosaccharide significantly increased population level of presumptive gut LAB in Oscar (*Astronotus ocellatus*).

### carnobacterium

Genus *Carnobacterium* belongs to the family Carnobacteriaceae within the order of Latobacillales and consists currently of 10 species of which; *Carnobacterium* (*piscicola*) *maltaromaticum, C. mobile, Carnobacterium divergens, C. alterfunitum*, and *C. inhibens* have been isolated from finfish intestine. The first study to isolate carnobacteria from GI tract of finfish, wild Atlantic salmon (*S. salar* L.), was carried out by Strøm (1988). She initially identified the bacterium as *Lactobacillus plantarum* Lab01, but later Ringø et al. (2001), reclassified the bacterium as *C. divergens*.

During the last 3 years, have several studies revealed genus *Carnobacterium* in finfish intestine (Table 1). As the distal intestine (DI) is considered to be the primary site of intestinal absorption of macromolecules in salmonids (Ringø et al., 2003; Desai et al., 2012), Lyons et al. (2017a) “investigated the diversity of allochthonous and autochthonous bacteria in DI of rainbow trout (*Oncorhynchus mykiss*) by next generation sequencing (NGS) and revealed that carnobacteria were the most prevalent of the autochthonous LAB genera (6.2%), and 4.15% of the allochthonous bacteria belonged to genus *Carnobacterium*.” In an investigation evaluated the dietary effect of black soldier fly (*Hermetia illucens*) by DGGE, Bruni et al. (2018) reported *Carnobacterium* sp., and that *C. divergens* were one of the dominant bacterial species in the insect-fed groups vs. control fed fish.

### lactobacillus

*Lactobacillus* are acid-tolerant facultative anaerobes, and they are either homo- or heterofermentative. Kraus (1961) carried out the first study revealing that fish, herring (*Clupea harengus* L.), contained lactobacilli in the GI tract. Since this pioneer study was carried out, have several reviews revealed *Lactobacillus* species in the GI tract of several finfish species (e.g., Ringø and Gatesoupe, 1998; Ringø, 2004; Ringø et al., 2005; Gatesoupe, 2008; Lauzon and Ringø, 2011; Merrifield et al., 2014).

Table 1 show that *Lactobacillus* spp., *Lb. aviarius, Lb. aviaries* subsp. *arafinosus, Lactobacillus brevis, Lb. crispatus/Lb. amylovorus, Lb. crispatus, Lb. collinoides, Lb. coryniformis, Lb. farciminis, Lb. gallinarum, Lb. johnsonii, Lb. reuteri*, and *Lb. sakei* have been reported in the GI tract of several finfish species during the last 3 years. Characterization of the DI microbiome of rainbow trout from both farm and aquarium settings were investigated by Lyons et al. (2016). Differences were noted in the microbial community within the intestine of both populations, Phylum Firmicutes was slightly more prominent in the aquarium reared fish, and within principal OTUs were identified as *Lactobacillus, Acetanaerobacterium, Catellicoccus, Streptococcus, Lactococcus, Leuconostoc, Enterococcus, Weissella*, and *Bacillus*. Bahramian and Parsa (2017) revealed that culturable *Lactobacillus* spp. was reduced in the GI tract of rainbow trout fed diets supplemented with essential oil of *Pistacia atlantica* subsp. *kurdica*. In the study of Lyons et al. (2017a), the authors revealed that *Lactobacillus* was present in very low abundance (0.1%), but a higher proportion (1.15%) of *Lactobacillus* was displayed by the allochthonous microbiota in the DI of rainbow trout.

An interesting topic within gut bacterial adherence and colonization is; to how increase the relative abundance of beneficial *Lactobacillus*. In a recent study, (Liu W. et al., 2017) evaluated the effect of gut adhesive *Lactobacillus* strains and the combined effect of short chain fucto-oligosaccharides (scFOS) on growth performance, gut adhesive bacteria and disease resistance of juvenile tilapia, and concluded that scFOS increased the relative abundance of the *Lactobacillus* strains.

The effect of chromic oxide (Cr_2_O_3_), one of the most widely used indicators for determination of nutrient digestibility in fish (Austreng, 1978; Ringø and Olsen, 1994), is less investigated in finfish studies. In three studies using Arctic charr (*Salvelinus alpinus* L.), Ringø (1993a,b, 1994) revealed that inclusion of 1% (Cr_2_O_3_) increased population level of culturable *Lactobacillus* and *Streptococcus*. In contrast, Serra et al. (2018) using the DGGE method to evaluate the gut microbiota of gilthead seabream (*Sparus aurata*) juvenile showed no effect of 0.5% inclusion level of Cr_2_O_3_ on number of operational taxonomic units, microbiota richness, diversity and similarity indices. The authors suggested that the difference between their results and Ringø's may be due to different inclusion level and the sharpening of the GI tract of the fish species.

### lactococcus

The genus *Lactococcus* is included within the family Streptococcacceae, and was described for the first time in 1985 after the division of genus *Streptococcus*, which included a group of microorganisms known as lactic streptococci represented by agents isolated from plant material, dairy cattle, and milk products (Schleifer et al., 1985). *Lactococcus* produce L (+) lactate from glucose as opposed to *Leuconostoc* produce D (–) lactate from glucose. One of the first studies isolating genus *Lactococcus* from finfish, common carp (*Cyprinus carpio*), was revealed by Cai et al. (1999), but later the genus has been isolated from the GI tract of several finfish species (Merrifield et al., 2014), and during the last years, numerous studies have revealed *Lactococcus* spp., *L. lactis garvieae, L. lactis* subsp. *cremoris, L. piscium*, and *L. raffinolactis* in the GI tract of finfish (Table 1). In their study with turbot (*Scophthalmus maximus*); autochthonous microbiota in the entire intestine, Yang et al. (2018) revealed that dietary stachyose significantly elevated the abundance of *Lactococcus* as well as *Carnobacterium, Pediococcus*, and *Enterococcus*. Li et al. (2015) used culture-dependent and culture-independent techniques to investigate the autochthonous bacterial communities in the whole intestine of grass carp (*Ctenopharyngodon idellus*) (Valenciennes) and revealed seven culturable strains showing high similarity (99%) to *L. raffinolactis* and one OUT similar to *L*. *raffinolactis*. Lyons et al. (2017a) revealed that both autochthonous and allochthonous *Lactococcus* was present in very low abundance (0.2 and 0.23%, respectively) in the DI of farmed rainbow trout.

### leuconostoc

*Leuconostoc* spp. are generally ovoid cocci often forming chains; are resistant to vancomycin and are catalase-negative. All *Leuconostoc* species are heterofermentative, produce D (–) lactate from glucose and are able to produce dextran from sucrose, and are generally slime-producers. Species of genus *Leuconostoc* are isolated from different sources (Carr et al., 2002) as well as from the GI tract of finfish (Merrifield et al., 2014). Since 2016, genus *Leuconostoc*, both autochthonous and allochthonous, has been reported in the intestine of rainbow trout, Atlantic salmon and Arctic charr (Table 1).

### pediococcus

*Pediococcus* usually occur in pairs or tetrads, and divide along two planes of symmetry, and they are purely homofermentative. To our knowledge, the first studies to isolate *Pediococcus* from intestine of finfish was carried out in the late 90's by Cai et al. (1999) and Halami et al. (1999). During the last 3 years, only one study has revealed *Pediococcus* in the intestine of finfish, turbot, evaluating the effect of dietary stachyose; a significant higher abundance of *Pediococcus* was revealed in fish fed diet added 5% stachyose (Yang et al., 2018).

### streptococcus

This genus has been subjected to important changes, as several species have been reclassified into genera *Lactococcus, Enterococcus*, and *Vagococcus*, based on biochemical characteristics and by molecular methods (Schleifer and Kilpper-Bälz, 1984; Schleifer et al., 1985; Collins et al., 1989). Species within genus *Streptococcus* have been isolated from several finfish species (Merrifield et al., 2014).

An overview of streptococci species revealed in the intestine of finfish since 2016 and until today is presented in Table 1. Lyons et al. (2017a) revealed that autochthonous *Streptococcus* was present in low abundance (2.3%) in the DI of farmed rainbow trout, but a slightly higher abundance (2.89%) was noticed by the allochthonous microbiota.

### enterococcus

Modern classification techniques of Enterococci resulted in the transfer of some members of genus *Streptococcus*, Lancefield's group D streptococci, to the new genus *Enterococcus*. Recently, Lyons et al. (2017a) revealed that autochthonous *Enterococcus* was present in low abundance (1.72%) in the DI of farmed rainbow trout. In addition to *Enterococcus* spp., *E. faecalis* and *Enterococcus faecium* were isolated from the GI tract of mrigal (*Cirrhinus mrigala*) (Shahid et al., 2017) and European sea bass (*Dicentrarchus labrax*) (Torrecillas et al., 2017), respectively.

### vagococcus

Collins et al. (1989) proposed that on the basis of the present sequence data and earlier chemotaxonomic studies that the motile group Lancefield group N cocci strains be classified in a new genus *Vagococcus*. The first study isolated *Vagococcus* (*Vagococcus fluvialis*) from finfish intestine was displayed by González et al. (2000). Recently Lyons et al. (2017a) revealed that autochthonous *Vagococcus* was present in low abundance (1.74%) in the DI of farmed rainbow trout, while the abundance of allochthonous *Vagococcus* was 0.72%.

### weissella

Genus *Weissella* belongs to Leuconostocaceae family and are obligate heterofermentative, producing CO_2_ from carbohydrate metabolism with either D (–), or a mixture of D (–)—and L (+)—lactic acid and acetic acid as major end products from sugar metabolism. According to the review of Fusco et al. (2015), there are 19 *Weissella* species known. The first study revealing *Weissella* (*W. confusa*) from the intestinal tract of fish, seabass (*Lates calcarifer*), was carried out by Rengpipat et al. (2014). During the last 3 years, several studies have revealed *Weissella* in the digestive tract of finfish (Table 1). For example, Lyons et al. (2017a) revealed that both autochthonous and allochthonous *Weissella* was present in very low abundance (0.1 and 0.39%) in the DI of farmed rainbow trout.

### bifidobacterium

*Bifidobacterium* are commonly reported in the GI tract of endothermic animals, but they are only been isolated in few studies from the digestive tract of finfish (Merrifield et al., 2014). Recently, Boonanuntanasarn et al. (2017) revealed increased population level of *Bifidobacterium* spp. by feeding Nile tilapia (*Oreochromis niloticus*) fingerlings fed inulin and Jerusalem artichoke (*Helianthus tuberosus*).

## Antibacterial effects of LAB; bacteriocins produced by LAB

Massive growth and intensification in aquaculture during the last decades has been associated with numerous problems; fish diseases caused by pathogenic bacteria being one of them (Sahoo et al., 2016). An array of conventional and advanced prophylactic or curative measures have been put forward to dispose of bacterial fish diseases, e.g., use of antibiotics (Burridge et al., 2010), vaccines (Gudding and Van-Muiswinkel, 2013), disinfectants, feed additives, dietary supplements, herbal immunostimulants (Newaj-Fyzul and Austin, 2014), prebiotics (Ganguly et al., 2012), and probiotics (e.g., Verschuere et al., 2000; Kesarcodi-Watson et al., 2008; Nayak, 2010; Pandiyan et al., 2013; Dawood and Koshio, 2016). The commonly use of disinfectants and antimicrobial agents as growth promotors and in disease control in aquaculture, increased the concern about the indiscriminate use due to the selective pressure on the intestinal microorganisms and development of antibiotic resistant bacteria (Cabello, 2006; Kolndadacha et al., 2011; Romero et al., 2012). As a natural consequence, there was seek for novel antibacterial compounds (preferably proteinaceous) with prophylactic or therapeutic potential and for which pathogens may not develop resistance (Patil et al., 2001; Sahoo et al., 2016).

The antibacterial agents are antibiotics, bacteriocins, lysozymes, proteases, siderophores, and/or hydrogen peroxide and acidic pH by organic acids production (De Vuyst and Leroy, 2007; Bindiya et al., 2015; Mukherjee et al., 2016).

Bacteriocins, are ribosomal-synthesized antimicrobial peptides, and LAB are the most common producers (Zacharof and Lovitt, 2012; Silva et al., 2018). They are small cationic molecules of 30–60 amino acids, form amphiphilic helices and are stable at 100°C for 10 min. During the last decade probiotic LAB with antimicrobial potential has achieved interest in aquaculture (Muñoz-Atienza et al., 2013), and the use of bacteriocins as supplements or adjuncts could be an eco-friendly approach to alleviate antibiotic overuse and resistance (Lagha et al., 2017).

Fish could be a potential source of bacteriocin-producing (bacteriocinogenic) bacteria and extensive screening of gut associated microorganisms may be taken up to avoid the use of antibacterial drugs in aquaculture (Sahoo et al., 2016). Reports indicated that the LAB isolated from diverse fish species, other aquatic organisms, culture water and sediments possess antagonistic activity against the fish pathogens (Balcázar et al., 2007a,b; Sugita et al., 2007; Ringø, 2008; Shahid et al., 2017). Hence, the potential use of bacteriocinogenic LAB as probiotics and bio-protective agents has received growing attention during the last decade (e.g., Gillor et al., 2008; Satish Kumar et al., 2011; Heo et al., 2012). According to Elayaraja et al. (2014), genera *Lactobacillus, Lactococcus, Streptococcus, Pediococcus, Oenococcus, Enterococcus, Leuconostoc*, and *Carnobacterium* produce a variety of bacteriocins. Numerous investigations on isolation and characterization of bacteriocins and bacteriocinogenic LAB from different sources are available, however, lesser research has been done on bacteriocins of LAB from fish (Gómez-Sala et al., 2015).

This section will present an overview on the beneficial attributes that might be associated with the use of bacteriocins and bacteriocinogenic LAB in aquaculture, diverse classes of bacteriocins produced by LAB, methods to characterize bacteriocins and an update on the efficacy of LAB against fish pathogens.

## Benefits associated with the LAB and bacteriocins produced by LAB

Interest on bacteriocinogenic bacteria, especially LAB, has achieved huge impetus due to its potential as both, probiotics and therapeutic antibiotics (Gillor et al., 2008; Cotter et al., 2013; Perez et al., 2014). Bacteriocins have several positive attributes that made them especially attractive for application in various sectors including aquaculture (Perez et al., 2014).

1. LAB and its metabolites are generally regarded as safe for human consumption, as they are found or used in food and fermented food products (FAO/WHO, 2002). Thus, aquatic organisms produced with application of LAB or bacteriocins thereof could be considered as safe for human consumption.
2. LAB bacteriocins are tolerant to high thermal stress and their activity over a wide pH range are well-known. Therefore, if applied as aquafeed supplement, efficacy of the bacteriocins from LAB is expected to be retained within the fish GI tract.
3. Bacteriocins forms pores in the target membrane of bacteria, even at extremely low concentrations.
4. These microbial metabolites are colorless, odorless, and tasteless, and therefore, do not interfere with acceptability of the diet if used as a supplement.
5. To our knowledge, there are no documentation on the development of resistant bacteria.
6. Bacteriocins usually have low molecular weight (rarely over 10 kDa), and they undergo posttranslational modification. Being proteinaceous, they can be easily degraded by the proteolytic enzymes of the host (Zacharof and Lovitt, 2012). Therefore, bacteriocin fragments do not live long either in the host or in the environment, thus minimizing the opportunity of target strains to interact with the degraded fragments and development of resistance.
7. Bacteriocins are ribosomally synthesized and produced during the primary phase of growth unlike antibiotics, which are usually secondary metabolites (Beasley and Saris, 2004). Bacteriocins generally restrict their activity to the strains of species closely related to the producer strain (Lisboa et al., 2006; Bakkal et al., 2012); compared to antibiotics having wider activity spectrum (broad-spectrum).
8. Not only antagonistic against some fish pathogens, bacteriocin has also been reported to be an important molecule in quorum sensing process (Czaran et al., 2002; Gobbetti et al., 2007). In fact, quorum sensing has been believed to be responsible for the expression of genes that code for bacteriocins in LAB. To outcompete the related species, sensing of its own growth enables the LAB to switch on bacteriocin production when competition for nutrients is likely to become more severe (Eijsink et al., 2002).

## Classes of bacteriocins produced by LAB

Gram-positive bacteria account for the majority of bacteriocins recorded *per se* (Rather et al., 2017), although bacteriocins are also revealed in Gram-negative (Sahoo et al., 2016). Among the Gram-positive bacteria, bacteriocins produced by LAB have gained particular attention nowadays. However, to deal with, firstly we need to see the classes of bacteriocins produced by diverse bacteria and then bacteriocins produced by LAB may be narrowed down.

Bacteriocin classification is an ongoing subject of debate, and therefore, proper classification is yet to be established (Desriac et al., 2010). A variety of criteria or their combinations are proposed as the basis for bacteriocin classification. For example, the producer bacterial family, molecular weights, amino acid composition, sequence homologies, primary structures, organization of the gene cluster (Hammami et al., 2010), mechanism of action and Gram designation. Bacteriocins were primarily divided into four classes (Klaenhammer, 1993). The Class I bacteriocins are called lantibiotics, represented by nisin and lactocin, gathers very low molecular weight (< 5 kDa) thermostable peptides, characterized by the post-translational modification and presence of lanthionine or derivatives. The Class II bacteriocins consist of small thermostable peptides (< 10 kDa) divided into three subclasses: IIa (pediocin and enterocin), IIb (lactocin G) and IIc (lactocin B). They are usually non-modified peptides, cationic, hydrophobic and often amphiphilic reflecting their ability to act on target cells by permeabilizing the cell membrane. Class IIa bacteriocins, the mostly studied LAB bacteriocins possessed strong antimicrobial properties against a broad range of Gram-positive spoilage and food-borne pathogens (Sahoo et al., 2016). The Class III bacteriocins having high molecular weight (>30 kDa), thermolabile peptides such as the helveticin J, while in the Class IV we can find large complexes of peptides with carbohydrates or lipids. Cotter et al. (2005) suggested a new classification; dividing bacteriocins into two categories: lantibiotics (Class I) and not containing lanthionine lantibiotics (Class II), while high molecular weight thermolabile peptides formally recognized under the above class III, would be separately re-classified as “bacteriolysins,” i.e., hydrolytic polypeptides. Thus, finally bacteriocins are divided into three major classes according to their genetic and biochemical characteristics (Drider et al., 2006). Consequently, different types of bacteriocins produced by the LAB are now classified (Table 2) as: Class I or Lantibiotics (<5 kDa), Class II or Non-Lantibiotics (usually <10 kDa) and Class III bacteriocins (generally > 30 kDa) (Ghosh et al., 2014).

**Table 2:** Different classes of bacteriocins produced by the LAB.

**Classes**	**Characteristic features**	**Bacteriocins produced**	**Typical producer organism**	**References**
Class I: Lantibiotics	Lantibiotics, small (<5 kDa) peptides containing lanthionine and b-methyllanthionine	Nisin, lactocin, mersacidin	*Lb. lactis* subsp. *lactis*	Parada et al., 2007
Class II: Non-lantibiotics	Small (<10 kDa), heat-stable, non-lanthionine-containing peptides			
Class IIa	Heat stable, non-modified, cationic, hydrophobic peptides; contain a double–glycine leader peptide; pediocin-like peptides	Pediocin PA1, sakicin A, leucocin A	*Lc. gelidum*	Todorov, 2009
Class IIb	Require synergy of two complementary peptides; mostly cationic peptides	Lactococcin G, plantaricin A, enterocin X	*E. faecium*	Perez et al., 2014
Class IIc	Affect membrane permeability and cell wall formation	Acidocin B, entereocin P, reuterin 6	*Lb. acidophilus*	Šušković et al., 2010
Class III: Large heat labile bacteriocins	Heat sensitive peptides, large molecular mass (>30 kDa)	Lysostaphin, enterolysin A, helveticin J	*Lb. helveticus*	Cotter et al., 2005

*Adapted and modified from Sahoo et al. (2016) and Mokoena (2017)*.

## Screening and characterization of bacteriocins produced by LAB

Bacteriocins are ribosomally synthesized peptides, which are usually synthesized as inactive precursors of peptides having an N-terminal sequence and later modified to attain an active state (Todorov, 2009; Perez et al., 2014). The activity of bacteriocins produced by different LAB is not uniform and constant, and depends on the physico-chemical composition of the microbial growth media (Balciunas et al., 2013). For aquaculture application of either bacteriocinogenic LAB or their bacteriocins, screening of efficient organism is a prerequisite. Bacteriocinogenic potential of a strain can be studied either by culture-dependent methods or by molecular methods employing PCR amplification of known bacteriocin structural genes. Initial screening to detect and determine the antibacterial activities of bacteriocinogenic strains can be done by an agar spot test (Schillinger and Lücke, 1989) or by agar well diffusion assay (Srionnual et al., 2007); using some indicator strains, e.g., *Lb. sakei* ssp. *sakei* JCM 1157T and *Listeria monocytogenes* ATCC 19111 (Lin et al., 2012). Then, antibacterial activity of the crude bacteriocin or bacteriocin like inhibitory substance (BLIS) may be further confirmed and optimized by characterization of the cell-free supernatants through pH and temperature adjustments, and proteinase-K treatment (Lin et al., 2012). For molecular detection of bacteriocinogenic potential, PCR amplification of known bacteriocin structural genes can be performed using the specific primers. For example, enterocin structural genes may be amplified with specific primers like EnterA-F/EnterA-R for detection of enterocin A (*entA*), EntB3/EntB5 for enterocin B (*entB*), EntP1/EntP2 for enterocin P (*entP*), and so on (Almeida et al., 2011; Gómez-Sala et al., 2015).

**Figure 1:**
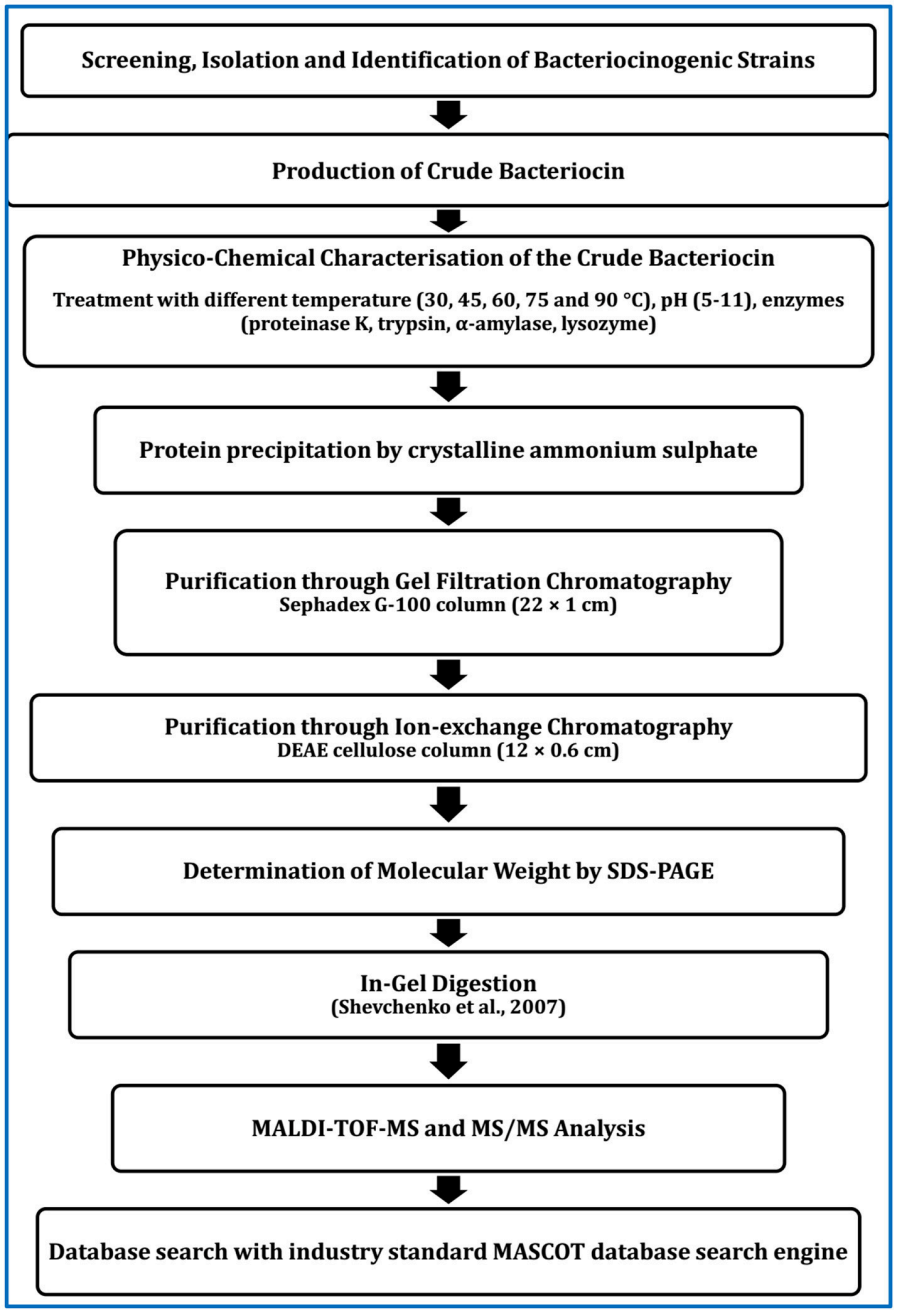
Scheme for purification of bacteriocins produced by LAB or other bacteria.

For application of bacteriocinogenic LAB as probiotics, screening and determination of potent LAB strain would be sufficient. However, for application of purified bacteriocin as feed supplement, production of pure bacteriocin and determination of molecular mass seem to be essential. Purification can be done by several steps as depicted in Figure 1: ammonium sulfate precipitation, gel filtration chromatography followed by ion-exchange chromatography. The active fraction that would display maximum antibacterial activity should be collected and used for further studies. The purity, homogeneity and molecular size of BLIS can be determined using sodium dodecyl sulfate-polyacrylamide gel electrophoresis (SDS-PAGE) (Srionnual et al., 2007). The molecular mass of the purified bacteriocin can be determined by matrix-assisted laser desorption/ionization time-of-flight (MALDI-TOF) mass spectrometry (MS) using a mass spectrometer and database search through Mascot search engine (Lin et al., 2012).

As per as aquaculture application is concerned, the use of purified bacteriocins is still a question mark, as the major apprehension would be administration of the compounds to the farmed fish that are aquatic. Numerous studies have recommended the bacteriocinogenic strains to be used as aquaculture probiotics (Irianto and Austin, 2002; Gatesoupe, 2008; Issazadeh et al., 2012). This is indeed a more reasonable and practical approach than direct application of purified bacteriocins in consideration of the fact that the probiotic strains are live cultures and thus able to ultimately establish themselves in the hosts and the aquatic environment (Rather et al., 2017).

## Activity of bacteriocinogenic LAB against fish pathogens: an update

It has been predicted that application of bacteriocins/BLIS from LAB or bacteriocinogenic LAB might not only effective in preventing diseases, but also minimize the risks of using broad-spectrum antibiotics in aquaculture. In aquaculture, numerous studies have indicated the potential use of bacteriocinogenic LAB as biocontrol agents against pathogens (e.g., Gillor et al., 2008; Desriac et al., 2010; Satish Kumar et al., 2011; Heo et al., 2012). Apart from LAB of fish origin, LAB from non-fish sources has also been tested to accomplish health benefits or disease prevention and achieved experimental success. For example, administration of the human probiotic, *Lactobacillus rhamnosus* 53101, reduced mortalities from 52.6 to 18.9% (10^9^ cells/g of feed) and to 46.3% (10^12^ cells/g of feed) in rainbow trout following challenge with *Aeromonas salmonicida* (Nikoskelainen et al., 2001). Furthermore, LAB-produced bacteriocins have been applied as bio-preservatives in marine food products and have shown to control pathogenic and spoilage microorganisms (Calo-Mata et al., 2007; Yin et al., 2007; Diop et al., 2009; Chahad et al., 2012).

To avoid harmful effects on the host fish as well as on the indigenous microbiota, use of autochthonous bacteria or their metabolites might be preferred to use vs. allochthonous. In aquaculture, the justification of using LAB or bacteriocinogenic LAB isolated from the autochthonous microbiota is based on the fact that the producer bacterial strains occupy more or less the same ecological niche with the pathogens and hosts of concern (Prasad et al., 2005; Zai et al., 2009). Antagonistic activity of LAB isolated from fish intestine against fish pathogens i.e., furunculosis, columnaris, peduncle disease, streptococcosis have been documented (e.g., Gutowska et al., 2004; Ringø et al., 2005; Sugita and Ito, 2006; Sahoo et al., 2016; Banerjee and Ray, 2017). Although, bacteriocins characterized from fish—and aquatic bacteria are scarce (Table 3), most of the characterized bacteriocins of aquatic origin that have antagonistic activity against many bacterial pathogens are isolated from marine aquaculture, while few from freshwater (Sahoo et al., 2016).

**Table 3:** Bacteriocins from LAB characterized and identified from aquatic resources.

**Bacteriocinogenic LAB**	**Source**	**Bacteriocin/BLIS (Molecular weight)**	**Antagonistic to pathogens**	**References**
*E. faecium*	Mangrove	Enterocin (5 kDa)	*Listeria monocytogenes, Lb. plantarum, Listeria innocua, E. faecalis, Salmonella typhi, Salmonella paratyphi*	Annamalai et al., 2009
*E. faecium* ALP7 *P. pentosaceus* ALP57	Marine shellfish	Enterocin B P Ediocin PA-1/AcH (<10 kDa)	*Listeria innocua, Listeria monocytogenes, Staphylococcus aureus, Bacillus cereus*, other LAB	Pinto et al., 2009
*Lb. acidophilus*	Gut of marine prawn (*Penaeus monodon*)	Bacteriocin (2.5 kDa)	*Lb. bulgaricus, Salmonella enteric* serovar *typhimurium, Staphylococcus aureus, Bacillus subtilis, Salmonella enterica* serovar *paratyphi* ‘B', *Escherichia coli, Klebsiella* sp., *Serratia marcescens, Pseudomonas aeruginosa*	Karthikeyan and Santhosh, 2009
*E. faecium* PE2-2	Sword fish	Enterocin A	*Listeria* sp., *Enterococcus* sp., *Staphylococcus* sp.	Valenzuela et al., 2010
*Lb. lactis*	Marine sediments (Chennai Harbor, India)	Bacteriocin (94 kDa)	*B. subtilis, Staph. aureus, E. faecalis, P. aeruginosa*	Rajaram et al., 2010
*E. faecium* MC13	Gray mullet (*Mugil cephalus*)	Enterocin (2.148 kDa)	*V. parahaemolyticus, Vibrio harveyi, A. hydrophila*	Satish Kumar et al., 2011
*Lb. fermentum*	Gray mullet (gut), prawn *(Penaeus monodon*) (muscle)	Bacteriocin (18 kDa)	*V. parahaemolyticus, L. monocytogenes, Listeria* sp., *Staph. aureus*	Indira et al., 2011
*L. lactis* PSY2	Marine perch fish (*Perca flavescens*)	Bacteriocin PSY2	*Arthrobacter* sp., *Acinetobacter* sp., *Bacillus subtilis, E. coli, L. monocytogenes*	Sarika et al., 2012
*E. thailandicus* B3-22	Gray mullet	BLIS (6.3 kDa)	*L. garvieae*	Lin et al., 2012
*Lactobacillus casei* AP8	Persian sturgeon (*Acipenser persicus*) (gut)	BLIS AP8 (5 kDa)	*E. coli, Listeria* spp., *Salmonella* spp., *Staph. aureus, A. hydrophila, V. anguillarum, B. cereus*	Ghanbari et al., 2013
*Lb. plantarum* H5	Persian sturgeon (gut)	BLIS H5(3 kDa)	*E. coli, Listeria* spp., *Salmonella* spp., *Staph. aureus, A. hydrophila, V. anguillarum, B. cereus*	Ghanbari et al., 2013
*Lb. brevis* FPTLB3	Mrigala (*Cirrhinus mrigala*)	BLIS (54 kDa)	*E. coli, E. faecalis, Lb. casei, Lb. sakei, Staph. aureus*	Banerjee et al., 2013
*Lb. fermentum* strain SBS001	Estuarine water	Bacteriocin (78 kDa)	*Klebsiella oxytoca, P. aeruginosa, E. coli*	Singh et al., 2013
*E. faecalis*	Marine environment	Bacteriocin (94 kDa)	*E. faecalis, Staph. aureus, B. subtilis*	Vadanasundari et al., 2013
*Lb. murinus* AU06	Marine sediments	BLIS (21 kDa)	*Micrococcus* sp., *Staph. aureus, P. aeruginosa, E. coli*	Elayaraja et al., 2014
*L. lactis* PSY2	Mucus and scales of marine fish (viz., *Platax* sp., *Perca* sp. and *Tuna* sp.)	Bacteriocin PSY2	*L. monocytogenes*	Sarika et al., 2017

It has been predicted that Pscicocin V1a and Pscicocin V1b isolated from *C. piscicola* CS526 and *C. piscicola* V1, respectively could prevent haemorrhagic septicaemia caused by *Pseudomonas* sp. (Bhugaloo-Vial et al., 1996). In another report, Phocaecin PI80 bacteriocin produced by *Streptococcus phocae* PI80 isolated from the gut of Indian white shrimp (*Peneaus indicus*) has been documented that might prevent *Vibrio* septicaemia caused by *Vibrio* sp. (Kumar and Arul, 2009). Likewise, BLIS AP8 from *Lactobacillus casei* AP8 and bacteriocin like inhibitory (substance) H5 from *Lb. plantarum* H5 might be effective against haemorrhagic septicaemia and *Vibrio* septicaemia (Ghanbari et al., 2013), although their mode of action is yet to be confirmed. In addition, the bacteriocin-producing LAB from aquatic organisms including fish include enterocin P produced by *E. faecium* isolated from turbot (Arlindo et al., 2006), nisin F produced by *L. lactis* from freshwater catfish (*Clarias gariepinus*) (De Kwaadsteniet et al., 2008), and divercins and piscicocins produced by *Carnobacterium* spp. (Desriac et al., 2010).

Although several reports have shown promising results regarding the aquaculture potential of bacteriocinogenic LABs or their bacteriocins from aquatic sources, subsequent studies are still needed to substantiate its viability in field condition with large number of organisms (Rather et al., 2017). Moreover, application strategy of the bacteriocins from LAB maintaining its effectiveness should be standardized so as to explore its potential in the disease prevention and sustainability of the aquaculture industry.

## LAB as probiotic

During the last years, numerous LAB strains have been used as probiotics in finfish aquaculture due to their health beneficial effect (Table 4). According to Belicova et al. (2013) an organism should be defined as probiotic when it is non-pathogenic, reveal antibacterial activities toward potential pathogens, tolerate low pH, high concentrations of conjugated, and de-conjugated bile salts, be accepted by the immune system, and not result in formation of antibodies. In addition, the probionts must not transfer antibiotic resistance genes to pathogens through horizontal gene transfer.

**Table 4:** An overview on LAB used as probiotic in finfish aquaculture.

**Probiotic**	**Doses and administrationduration**	**Fish species**	**Parameters examined**	**References**
*Lb. plantarum*	2 × 10^7^ CFU g^−1^–72 days	Rainbow trout (*Oncorhynchus mykiss*)	Growth performance and immune parameters	Soltani et al., 2017b
	10 × 10^9^ CFU/kg–90 days	European sea bass (*Dicentrarchus labrax*)	Growth performance and serum biochemical parameters	Piccolo et al., 2015
	10^8^ CFU g^−1^–60 days	Rainbow trout	Serum biochemical as well as immune responses	Kane et al., 2016
	1.2 × 10^6^, 0.9 × 10^6^, and 0.56 × 10^6^ cfu/g–80 days	Common carp (*Cyprinus carpio*)	Growth performance, Immune parameters, disease resistance	Soltani et al., 2017a
	1.81 × 10^7^ CFU g^−1^–58 days	Nile tilapia	Growth performance, haemato-immunological parameters and gut microbiota	Yamashita et al., 2017
	10^8^ CFU g^−1^–28 days	Nile tilapia	Intestinal microbiota, growth performance and resistance against Cd exposure	Zhai et al., 2017
			Growth performance and resistance against waterborne aluminum exposure	Yu et al., 2017
Heat killed *Lb. plantarum*	0.01, 0.1, 1 and 2 g kg^−1^–56 days	Red sea bream (*Pagrus major*)	Growth performance, immune parameters and antioxidant defense	Dawood et al., 2015
*Lb. plantarum + B. subtilis + P. aeruginosa*	0.5 × 10^8^ CFU g^−1^–60 days	Rohu (*Labeo rohita*)	Immune parameters, antioxidant defenses and disease resistance	Giri et al., 2014
*Lb. plantarum + L. lactis*	log_10_ 7.0 CFU/g–30 days	Olive flounder (*Paralichthys olivaceus*)	Immune parameters and disease resistance	Beck et al., 2015
*Lb. plantarum +*LMWSA	10^8^ CFU g^−1^–60 days	Nile tilapia (*Oreochromis niloticus*)	Growth performance, immune parameters and disease resistance	Van Doan et al., 2016c
*Lb. plantarum +* Jerusalem artichoke	10^8^ CFU g^−1^–12 weeks	Pangasius catfish (*Pangasius bocourti*)	Growth performance, immune parameters and disease resistance	Van Doan et al., 2016a
*Lb. plantarum +* Eryngii mushroom (*Pleurotus eryngii*)	10^8^ CFU g^−1^–90 days	Pangasius catfish	Growth performance, immune parameters and disease resistance	Van Doan et al., 2016b
*Lb. acidophilus*	1.5 × 10^8^, 3 × 10^8^ and 6 × 10^8^ CFU g^−1^–70 days	Black swordtail (*Xiphophorus helleri*)	Growth performance, mucosal immunity and intestinal microbiota	Hoseinifar et al., 2015c
	1.5 × 10^8^, 3 × 10^8^ and 6 × 10^8^ CFU g^−1^–56 days	Gold fish (*Carassius auratus gibelio*)	Skin mucus protein profile and immune parameters, appetite and immune related genes expression	Hosseini et al., 2016
	10^6^ CFU g^−1^–15 days	Nile tilapia	Immune related genes expression and disease resistance	Villamil et al., 2014
*Lb. acidophilus + B. cereus* + *Clostridium butyricum*	1.0 × 10^9^ CFU g^−1^–60 days	Hybrid grouper (*Epinephelus lanceolatus*♂ × *Epinephelus fuscoguttatus* ♀)	Growth performance, digestive and antioxidant enzymes activities	He et al., 2017
*Lb. casei*	5 × 10^6^, 5 × 10^7^ and 5 × 10^8^ CFU g^−1^–60 days	Shirbot (*Barbus gryprus*)	Growth performance and digestive enzymes activity	Mohammadian et al., 2017
	1.0 × 10^8^ cells/g–28 days	Zebrafish (*Danio rerio*)	Reproductive performance and related genes expression	Qin et al., 2014
*Lb. casei +* apple cider vinegar	10^8^ CFU g^−1^–56 days	Common carp	Serum and mucus immune parameters, immune and antioxidant defense related genes expression	Safari et al., 2017
*Lb. paracasei*	10^6^ CFU g^−1^–66 days	Rainbow trout	Growth performance and intestinal microbiota	Lopez Cazorla et al., 2015
*Lb. delbrueckii*	1 × 10^5^, 1 × 10^6^, 1 × 10^7^ and 1 × 10^8^ CFU g^−1^	Common carp	Intestinal immune parameters, immune related genes expression, antioxidant defense, disease resistance	Zhang C.-N. et al., 2017
*Lb. delbrueckii* ssp. *bulgaricus*	5 × 10^7^ CFU g^−1^–60 days	Shirbot	Immune parameters and disease resistance	Mohammadian et al., 2016
*Lb. rhamnosus*	10^3^,10^5^ and 10^6^ CFU/g–63 days	European eel (*Anguilla anguilla*)	Sperm quality and quantity, expression of genes related to spermatogenesis	Vílchez et al., 2015
	1 × 10^2^, 1 × 10^4^ and 1 × 10^6^ cells g^−1^–56 days	Red sea bream	Plasma and mucus parameters	Dawood et al., 2017
	10^7^ and 10^8^ CFU g^−1^–56 days	Rainbow trout	Intestinal microbiota and histology, biochemical parameters, and antioxidant defense	Topic (Popovic et al., 2017)
*Lb. rhamnosus+ Lb. lactis*	10^6^ × cell/g–56 days	Red sea bream	Immune parameters and antioxidant defense	Dawood et al., 2016b
*P. acidilactici*	10^6^ CFU/g–10 days	Zebrafish	Expression of genes related to male and sperm quality	Valcarce et al., 2015
	1 g kg^−1^–56 days	Green terror (*Aequidens rivulatus*)	Innate immune parameters and resistance to hypoxia stress	Neissi et al., 2013
*P. acidilactici+* galactooligosaccharide (GOS)	7.57 log CFU g^−1^–56 days	Rainbow trout	Growth performance, immune parameters and disease resistance	Hoseinifar et al., 2015a,b, 2016a
*P. acidilactici+* GOS	7.57 log CFU g^−1^–56 days	Common carp	Immune parameters and related genes expression	Modanloo et al., 2017
*P. pentosaceus*	6 × 10^10^, 1.6 × 10^11^, 1.6 × 10^12^ and 3.2 × 10^12^ cells g^−1^–56 days	Red sea bream	Skin mucus and serum immune parameters, resistance to low-salinity stress	Dawood et al., 2016a
	2 × 10^7^, 2 × 10^8^ and 2 × 10^9^ CFU g^−1^–56 days	Siberian sturgeon	Intestinal and body composition	Moslehi et al., 2016
	10^9^ CFU g^−1^–21 days	Orange-spotted grouper (*Epinephelus coioides*)	Growth performance, immune related genes expression and disease resistance	Huang J.-B. et al., 2014
*W. cibaria*	1.18 × 10^7^ CFU g^−1^–45 days	Brazilian native surubins	Growth performance, haemato-immunological parameters and intestinal histomorphology	Jesus et al., 2017
*Lc. mesenteroides+ E. faecalis+ Lb. fermentum*	10^5^, 10^7^ and10^9^ CFU g^−1^–56 days	Javanese carp *(Puntius gonionotus)*	Growth performance, intestinal microbiota and body composition	Allameh et al., 2016
*L. lactis* WFLU12	10^9^ CFU g^−1^–56 days	Olive flounder	Growth performance, immune parameters and disease resistance	Nguyen et al., 2017
*E. faecium*	10^7^ CFU/g–35 days	Javanese carp	Digestive enzymes activity, intestinal short chain fatty, disease resistance	Allameh et al., 2015
*E. gallinarum* L-1	10^6^, 10^7^, and 10^8^ cfu mL^−1^–28 days	Sea bream, European sea bass, meager (*Argyrosomus regius*) and red porgy (*Pagrus pagrus*)	Immune parameters and peroxidase content	Román et al., 2015
*E. casseliflavus*	10^7^, 10^8^, and 10^9^ CFU g^−1^–56 days	Rainbow trout	Intestinal microbiota, humoral immune parameters and disease resistance	Safari et al., 2016

Considering the potential of LAB as feed additive in aquaculture there is extensive literatures available. The researchers investigated possible effects on growth performance, feed utilization, digestive enzymes activity, immune response, and disease resistance. Despite some contradictory results, most of the studies revealed beneficial effects on measured parameters. This section present an overview on available literatures regarding LAB administration as probiotic in aquaculture. To avoid overlap with previous reviews, we have focused on the papers published from 2014. Readers with special interests on previous studies, are referred to the reviews of Ringø and Gatesoupe (1998), Nayak (2010), Carnevali et al. (2014), Castex et al. (2014), De et al. (2014), Lauzon et al. (2014), Merrifield et al. (2014), Ringø et al. (2014) and Hoseinifar et al. (2016c).

### *lactobacillus* spp.

#### lactobacillus plantarum

Within lactobacilli, *Lb. plantarum* is the most studied strain. Piccolo et al. (2015) evaluated the effects of dietary *Lb. plantarum* on performance and serum biochemical parameters of European sea bass. The inclusion level was 10 × 10^9^ CFU/kg and fishes were fed on the probiotic supplemented diet for 90 days and probiotic feeding revealed noticeable effect on growth performance vs. control. Regarding serum biochemical parameters only total cholesterol and triglycerides were studied, but a significantly increased following probiotic administration was revealed. In a 72-days feeding trial, Soltani et al. (2017a) fed rainbow trout (vaccinated to yersiniosis) a probiotic diet containing *Lb*. *plantarum*, 2 × 10^7^ CFU g^−1^. At the end of the trial, the vaccinated fish fed the probiotic diet had noticeably higher lysozyme and alkaline phosphatase compared to the other treatments. Besides, improved growth performance was noticed in the vaccine + probiotic treatment vs. the others. However, no significant difference among different treatments in case of hameato-immunological parameters as well as LAB levels in intestinal microbiota were revealed. The authors concluded that administration of probiotics following vaccination can be considered as beneficial by increasing vaccines efficacy. The same research group, Kane et al. (2016), evaluated the effects of 10^8^ CFU g^−1^ of *Lb. plantarum* on serum biochemical as well as immune responses in rainbow trout treated with streptococcosis/lactococosis vaccine, and revealed that feeding *Lb. plantarum* to immunized fish resulted in significant increase of immune parameters such as lysozyme, alternative complement activities, antibody titer, total leukocytes and lymphocytes, and serum biochemical parameters. Moreover, Soltani et al. (2017b) supplemented a common carp diet with different levels (1.2 × 10^6^, 0.9 × 10^6^, and 0.56 × 10^6^ CFU/g) of *Lb*. *plantarum*, and after 80 days feeding; significantly improved growth performance and immune parameters compared to the control treatment was noticed. However, probiotic administration had no significant effect on liver enzymes level. The challenge test showed that probiotic fed fish had higher resistance against *Aeromonas hydrophila*. When discussing the effect of probiotic toward disease resistance, Fečkaninová et al. (2017) reviewed and highlighted the potential of LAB to protect against different *Aeromonas* spp. in salmonid aquaculture.

The possible effects of *Lb*. *plantarum* on growth performance, haemato-immunological factors, intestinal microbiota and histology as well as disease of Nile tilapia was studied by Yamashita et al. (2017). Interestingly, dietary administration of *Lb*. *plantarum* increased LAB level and decreased Vibrionaceae counts in intestinal microbiota. Besides, feeding on probiotic improved growth performance and feed utilization, while no significant difference was observed pre-challenge, but probiotic fed fish showed improved hematological parameter post-challenge. On the other hand, histological evaluations, intestinal epithelium structure, revealed no significant difference between probiotic treatment and control fed fish. In a study using Nile tilapia, Zhai et al. (2017) evaluated the protective effects of 10^8^ CFU g^−1^ *Lb. plantarum* against cadmium exposure. The study included four treatments; control, probiotic, Cd exposure and Cd exposure + probiotic. The exposure with Cd drastically decreased the richness of intestinal microbiota. However, feeding with probiotic reversed the changes were revealed. In addition, the highest growth performance was noticed in fish fed probiotics. The protective effects of *Lb*. *plantarum* against waterborne aluminum exposure of tilapia by Yu et al. (2017), revealed that fish fed *Lb. plantarum* CCFM639 significantly increased growth performance and alleviated aluminum damages. The effect of different levels of *Lb*. *plantarum* (1 × 10^7^, 1 × 10^8^, and 1 × 10^9^ CFU g^−1^) on growth performance and immune parameters in Siberian sturgeon (*Acipenser baerii*) were investigated by Pourgholam et al. (2016). Compared to control treatment, significant increase of innate immune parameters were noticed in probiotic fed fish, and the highest level of immunity was observed in fish fed 1 × 10^8^ CFU g^−1^ probiotic as well as improvements of growth performances.

Dietary administration of head-killed probiotic has been suggest as efficient and safe feed additive in aquaculture (Yan et al., 2016). Beside working on live *Lb*. *plantarum*, the efficacy of dead *Lb*. *plantarum* was evaluated by Dawood et al. (2015). Red sea bream (*Pagrus major*) with average weight of 11g were fed different levels (0.01, 0.1, 1, and 2 g kg^−1^) of heat killed *Lb. plantarum* for 56 days. The results revealed improved growth performance, immune parameters as well as antioxidant defense. The author displayed that 1 g kg^−1^ was the best inclusion level of heat killed *Lb. plantarum* for Red sea bream. However, as there is limited information available on the use of dead or inactivated probiotics on other species, this topic merits further investigations.

A review of the literature showed that, *Lb. plantarum* has been used as multi-strain probiotic and in combination with *Bacillus subtilis* VSG1 and *Pseudomonas aeruginosa* VSG2 (Giri et al., 2014), and feeding rohu (*Labeo rohita*) a multi-strain probiotic supplemented diet increased immune parameters, antioxidant defenses as well as disease resistance. The study also revealed that multi-strain administration was more efficient than single administration. In a study with olive flounder (*Paralichthys olivaceus*) fed *Lb. plantarum* FGL0001 and *Lac. lactis* BFE920 as multi-strain probiotics (Beck et al., 2015), the authors observed higher immune parameters and disease resistance in fish fed multi-strain probiotic vs. individual probiotic.

Gibson and Roberfroid (1995) proposed the synbiotics (a combination of pro- and prebiotics) concept; “*characterize some colonic foods with interesting nutritional properties that make these compounds candidates for classification as health-enhancing functional ingredients*.” This concept is well used in endothermic studies (e.g., DuPont and DuPont, 2011; Ford et al., 2014) as well as in fish (Ringø and Song, 2016). Van Doan et al. (2016a) evaluated combined administration of low molecular weight sodium alginate (LMWSA) as prebiotic with *Lb. plantarum* in Nile tilapia diet, and concluded that co-application increased the immunomodulatory effect as well as disease protecting effects of *Lb. plantarum*. Similar results were observed when Jerusalem artichoke (Van Doan et al., 2016b) or Eryngii mushroom (*Pleurotus eryngii*) (Van Doan et al., 2016c) were used in combination with Lb. plantarum in a diet fed to Pangasius catfish (*Pangasius bocourti*).

#### lactobacillus acidophilus

*Lactobacillus acidophilus* has been used as common probiotic in aquatic animals. Hoseinifar et al. (2015c) addressed the effects of different dose of *Lb. acidophilus* (1.5 × 10^8^, 3 × 10^8^, and 6 × 10^8^ CFU g^−1^) on intestinal microbiota, mucosal immune parameters as well as stress resistance in black swordtail (*Xiphophorus helleri*). At the end of feeding trial, the probiotic strain successfully colonized the intestine and the dose of LAB significantly increased. Probiotic treatment, also increased growth performance as well as skin mucus immunity. Swordtail fish fed with *Lb. acidophilus* showed significantly higher resistance when exposed to salinity stress test. In another study with ornamental fish, Hosseini et al. (2016) investigated possible effects of *Lb. acidophilus* as probiotic on protein profile and immune parameters of skin mucus as well as ghrelin gene expression of gold fish (*Carassius auratus gibelio*). Dietary probiotic affected protein profile and improved immune parameters. Interestingly, feeding on probiotic suppressed appetite related gene, while, immune related genes were up-regulated by probiotic treatments. These studies highlighted the potential of *Lb. acidophilus* as probiotic for ornamental fish.

Furthermore, in a study with Nile tilapia, Villamil et al. (2014) evaluated possible effects of *Lb. acidophilus* on the expression of immune related genes as well as resistance against *A. hydrophila*. The results showed up-regulation of IL-1β and transferrin in spleen and kidney. Also, feeding on probiotic supplemented diet resulted in higher protection against disease. Furthermore, the author reported that extracellular products (ECPs) of *Lb. acidophilus* inhibited the growth of different fish pathogens under *in vitro* conditions. He et al. (2017) carried out a study on hybrid grouper (*Epinephelus lanceolatus*♂ × *Epinephelus fuscoguttatus* ♀) fed either single *Lb. acidophilus* LAG01 or in combination with *B. cereus* BC-01, *Clostridium butyricum* CBG01 for 60 days. Feeding on either *Lb. acidophilus* or combination of three strains remarkably increased growth performance. Similar results were observed in case of digestive- and antioxidant enzymes activities. However, no statistical significant difference were revealed between mono or multi-strain probiotic supplementation.

#### lactobacillus casei

In a 60-days feeding trial with shirbot (*Barbus gryprus*) fed four experimental diets with varying dose (5 × 10^6^, 5 × 10^7^, and 5 × 10^8^ CFU g^−1^) of *Lb. casei*, the results revealed higher performance in probiotic fed fish (Mohammadian et al., 2017). Furthermore, chymotrypsin and trypsin activities in probiotic groups were remarkably higher compared to the control. Safari et al. (2017) showed beneficial effects of *Lb. casei* on innate immune parameters (either serum or skin mucus) as well as expression of selected immune and antioxidant defense related genes. Moreover, the authors revealed that combined administration of probiotic with apple cider vinegar improved efficacy of the probiotic supplementation. This study highlighted the importance of additional research on evaluation of other feed additives (e.g., medicinal herbs and prebiotics) to be used in combination with probiotics, a topic being less investigated in fish (Ringø and Song, 2016).

Zebrafish (*Danio rerio*) has been suggested as model organism in human and animal studies (Penberthy et al., 2002; Hoseinifar et al., 2017). The possible effects of *Lb. casei* as probiotic on reproductive performance and maternal immunity of zebra fish was studied by Qin et al. (2014). Zebrafish fed the probiotic diet for 28 days displayed remarkably improved reproductive parameters such as egg ovulation, fertilization, and hatching rate. Furthermore, feeding on probiotic noticeably increased the expression of selected genes related to reproduction (*eptin, kiss2, gnrh3, fsh, lh, lhcgr*, and *paqr8*).

#### lactobacillus paracasei

In a study using rainbow trout (31.25 ± 3.43 g), Lopez Cazorla et al. (2015) tested *Lb. paracasei* subsp. *tolerans* F2 as probiotic on growth performance and intestinal microbiota. This probiotic was originally isolated from the digestive tract of *Ramnogaster arcuate* (Osteichthyes, Clupeidae). The results revealed significant effects on growth performance parameters and LAB dose in intestinal microbiota of probiotic fed fish was significantly higher vs. control.

#### lactobacillus delbrueckii

The effects of dietary *Lb. delbrueckii* (1 × 10^5^, 1 × 10^6^, 1 × 10^7^, and 1 × 10^8^ CFU g^−1^) on immune parameters as well as protection against *A. hydrophila* in carp was studied by Zhang C.-N. et al. (2017) and revealed improved intestinal immune parameters. Furthermore, probiotic feeding affected immune related genes expression; down-regulation of *TNF-*α*, IL-8, IL-1*β*, and NF-*κ*Bp65* and up-regulation of *IL-10* and *TGF-*β genes. Moreover, fish fed with 1 × 10^6^ CFU g^−1^ *Lb. delbrueckii* showed increased antioxidant defense both at gene expression and enzyme levels. The challenge test showed higher protection against *A. hydrophila* infection. Mohammadian et al. (2016) used a *Lb. delbrueckii* ssp. *bulgaricus* isolated from shirbot intestine and supplemented the diet with the probiotic at rate of 5 × 10^7^ CFU g^−1^. At the end of feeding trial, 60 days, immune parameters as well as resistance against *A. hydrophila* were measured. Evaluation of immune response and disease resistance revealed higher immune parameters (lysozyme, complement, and respiratory burst activities) as well as survival rate after challenge test (Mohammadian et al., 2016).

#### lactobacillus rhamnosus

In a study using European eel (*Anguilla anguilla*), Vílchez et al. (2015) administered three dose (10^3^,10^5^, and 10^6^ CFU/g) of *Lb. rhamnosus* in the diet and monitored possible effects on spermatogenesis process. After 63 days of oral administration, up-regulation of genes related to reproduction such as *activin*, androgen receptors α and β (*ar*α and *ar*β), progesterone receptor 1 (*pr1*), bone morphogenetic protein 15 (*bmp15*), and FSH receptor (*fshr*) was noticed. These changes at molecular levels were corresponded with observed changes in sperm quality and quantity. The authors concluded that *Lb. rhamnosus* confers the spermatogenesis process in European eel. Dawood et al. (2016b) also conducted an investigation on the effects of *Lb. rhamnosus* (either single e or combined with *Lb. lactis*) on growth performance and immune parameters of red sea bream, and displayed increased immune parameters and antioxidant defense in fish fed supplemented diet; higher effect was revealed when the two strains was used simultaneously. Similar effects were observed on growth performance and feed utilization. Moreover, probiotic administration decreased total cholesterol and triglycerides levels. The same research group, evaluated in another study the effects of varying dose (1 × 10^2^, 1 × 10^4^, and 1 × 10^6^ cells g^−1^) of *Lb. rhamnosus* on red sea bream (Dawood et al., 2017), showed significant increase of plasma and mucus parameters (total protein, mucus myeloperoxidase activity, and mucus secretion), and concluded the results to be a sign for beneficial effects on host physiological responses. In a study with rainbow trout, Popovic et al. (2017) investigated the effect of dietary *Lb. rhamnosus* (10^7^ and 10^8^ CFU g^−1^) on intestinal microbiota and histology, biochemical parameters, and antioxidant defense in a 6-weeks feeding trial. While probiotic feeding had no significant effects on antioxidant defense, biochemical parameters were affected. Moreover, histological investigations revealed improvement of microvilli length in the proximal intestine (PI) as well as enhanced number of goblet cells in PI and distal intestine of probiotic fed fish. The authors concluded that *Lb. rhamnosus* was a promising feed additive, capable of improving rainbow trout health (Popovic et al., 2017).

### *pediococcus* spp.

#### pediococcus acidilactici

During the past years there was increasing interests toward administration of *Pediococcus* spp. as probiotic in aquaculture and most of the studies have focused on *P. acidilactici*; the commercial product named Bactocell. For instance, the possible effects of dietary *P. acidilactici* (10^6^ CFU/g) was assessed on sperm quality in zebrafish (Valcarce et al., 2015). After 10 days treatment of zebrafish male with probiotic, remarkable up-regulation of selected genes related to male and sperm quality was noticed. Hoseinifar et al. (2015a,b, 2016b) studied the effects of single or combined administration of *P. acidilactici* and galactooligosaccharide in rainbow trout. While single administration had no significant effects on growth performance, combined administration remarkably improved growth performance parameters. Also, feeding on supplemented diet remarkably increased immune response and resistance against *Streptococcus iniae*. Similar results were observed in a study using common carp (Modanloo et al., 2017). Furthermore, in a study with ornamental fish, green terror (*Aequidens rivulatus*), Neissi et al. (2013) studied the effects 0.1% inclusion of commercial *P. acidilactici* and revealed remarkable increase of the innate immune parameters as well as improvement of stress indicators following exposing fish to hypoxia stress.

#### pediococcus pentosaceus

Recently, *Pediococcus pentosaceus* has received attention as probiotic, but still limited information on the use of this strain is available. In a 56 days study, the effects of different dose (1.6 × 10^10^, 1.6 × 10^11^, 1.6 × 10^12^, and 3.2 × 10^12^ cells g^−1^) of inactivated *P. pentosaceus* was evaluated in red sea bream (Dawood et al., 2016a). Dietary administration of inactivated probiotic noticeably increased growth performance as well as mucus secretion. Also, skin mucus and serum immune parameters showed increment following treatment with probiotic. Furthermore, fish fed the probiotic supplemented diets had remarkably higher low-salinity stress resistance. Based on these results the authors suggested that inactivated *P. pentosaceus* as efficient and safe probiotic. Likewise, Moslehi et al. (2016) reported modulation of intestinal microbiota as well as body composition in Siberian sturgeon following dietary administration of a *P. pentosaceus* strain isolated from Persian sturgeon intestine. Furthermore, Huang J.-B. et al. (2014) addressed the effect of *P. pentosaceus* as probiotic in orange-spotted grouper (*Epinephelus coioides*). The probiotic bacteria was originally isolated by the authors from cobia (*Rachycentron canadum*) intestine. The strain showed antagonistic effects against pathogens under *in vitro* conditions and in an *in vivo* experiment, dietary administration of *P. pentosaceus* significantly increased growth performance, immune related genes expression as well as disease resistance.

### *weissella* spp.

There is relatively limited information available about efficacy of *Weissella* species as probiotic in aquaculture. In recent study with Brazilian native surubins (43.3 g), the effects of dietary *Weissella cibaria* (1.18 × 10^7^ CFU g^−1^) was investigated on performance, haemato-immunological parameters and intestinal histomorphology (Jesus et al., 2017). Regarding the hematological parameters, most of the parameters remained unaffected, except red blood cells, thrombocyte and lymphocyte counts which were higher in probiotic fed fish. Evaluation of immune parameters revealed higher phagocytosis, agglutination titer, and total Ig in probiotic groups compared with control. Feeding on probiotic supplemented diets significantly improved intestinal histology as observed increased height and width and number of villi as well as mucus producing goblet cells counts per villi. These results highlighted the potential of *Weissella* spp. to be used as a novel probiotic in aquaculture.

### *leuconostoc* spp.

To our knowledge, possible effects of *Leuconostoc* as probiotic has only been investigated in one study. Allameh et al. (2016) supplemented Javanese carp (*Puntius gonionotus*) diet with either single *Lc. mesenteroides* or in combination with *E. faecalis* and *Lb. fermentum* as multi-strains probiotics. Interestingly, growth performance of fish fed single *Lc. mesenteroides* was better than those fed multi-strains probiotic. However, no significant effect was noticed in body composition.

### *lactococcus* spp.

Nguyen et al. (2017) isolated *L. lactis* WFLU12 from intestine of wild marine fishes and based on *in vitro* probiotic effects selected the strain to be used in olive flounder diet. Interestingly, inclusion of this host-associated probiotic caused improvement of immune responses and protection against *Streptococcus parauberis* infection. Besides, probiotic fed fish showed improved growth performance and feed utilization. These results highlighted the importance of isolation of host-associated probiotic, a topic that merits further investigations.

### *enterococcus* spp.

*Enterococcus* spp. and especially *E. faecium* are among the most studied probiotics in aquaculture, and from 2014 there are some reports available. For instance, Allameh et al. (2015) studied possible effect of oral administration of *E. faecium* on physiological responses of Javanese carp. Fish were fed on a single dose (10^7^ CFU/g) for 5 weeks and at the end of the rearing period; significant increase of digestive enzymes activity as well as intestinal short chain fatty acid production (propionic and butyric acid) were noticed in the probiotic group. These improvements were in line with increased protection against *A. hydrophila* challenge. In accordance, elevation of cell-mediated immune response following oral administration of *E. faecium* has been reported by Matsuura et al. (2017). Besides the results on *E*. *faecium*, there are interests toward other species of this genus. *Enterococcus gallinarum* L-1 was used as potential probiotic in different species including gilthead sea bream, European sea bass, meager (*Argyrosomus regius*) and red porgy (*Pagrus pagrus*) diets (Román et al., 2015). The strain was originally isolated from gilthead sea bream intestine and the authors tested different forms; live or inactivated with heat or U.V. The authors reported no immunostimulatory effects of *E. gallinarum* in meager, however, immune stimulation was noticed in sea bream, sea bass and red porgy leucocytes. The immunostimulatory effects were increased along with elevation of probiotic level in diet; highest dose in the 10^8^ CFU mL^−1^ treatment. Furthermore, Safari et al. (2016) isolated *Enterococcus casseliflavus* from rainbow trout intestine and evaluated its probiotic potential in rainbow trout. The probiotic strain was orally administered at rate of 10^7^, 10^8^, and 10^9^ CFU g^−1^ for 8 weeks. At the end of feeding trial, significant change was noticed in LAB counts in the intestinal microbiota. This change was in line with remarkably increase of humoral immune parameters. Also, probiotic fed fish had significantly higher resistance when exposed to experimental challenge with *S. iniae*. Based on these results the authors suggested this host-associated strain as beneficial probiotic for rainbow trout culture.

When discussing the use of probiotics, it is of interest to notice that *Lb. rhamnosus* GG outcompete vancomycin-resistant *E. faecium* via mucus-binding pili (Tytgat et al., 2016), and the finding of He et al. (2017) using *Lb. rhamnosus* GG and its mutant (PB22) lacking SpaCBA pili to investigate the influence of pili on spatial distribution. LGG showed a mucosa type distribution, while PB22 revealed a hybrid distribution and the disease protection was accordingly improved.

However, prior to use of probiotics; injury to the mucosa and epithelial cells should be investigated in details as *Lb. plantarum* originally isolated from traditional Sabalan Iranian cheese from sheep raw milk resulted in damaged epithelial cells and disorganized microvilli of beluga (*Huso huso*) (Salma et al., 2011), while LGG induced injury to the mucosa of zebrafish (He et al., 2017).

## Pathogenic LAB

In addition to probiotic, some pathogenic LAB are also documented (Ringø and Gatesoupe, 1998; Leisner et al., 2007; Michel et al., 2007). This sub-chapter focus on pathogenic LAB in aquaculture (Table 5), and the treatments (Table 6).

**Table 5:** Pathogenic LAB in aquaculture.

**Pathogenic LAB species**	**Studied species**	**References**
*S. agalactiae*	Silver pomfret (*Pampus argenteus*) Red tilapia (*Oreochromis niloticus*) Golden pompano (*Trachinotus blochii*) Barcoo grunter (*Scortum barcoo*) Hybrid tilapia (*O. niloticus* × *O. aureus*)	Duremdez et al., 2004 Musa et al., 2009 Amal et al., 2012 Liu et al., 2014 Al-Harbi, 2016
*S. iniae*	Hybrid striped bass (*Morone chrysops* × *Morone saxatilis*) Rabbitfish (*Siganus canaliculatus*) Sea bass (*Dicentrarchus labrax*) Japanese flounder (*Paralichthys olivaceus*) Barramundi (*Lates calcarifer*) Hybrid tilapia	Stoffregen et al., 1996 Yuasa et al., 1999 Colorni et al., 2002 Nguyen et al., 2002 Bromage et al., 1999 Al-Harbi, 2011
*S. dysgalactiae* *S. parauberis* *S. uberis*	Sturgeon (*Acipenser schrenckii*) Wild striped bass (*Morone saxatilis*) Mandarin fish (*Siniperca chuatsi*)	Yang and Li, 2009 Haines et al., 2013 Luo et al., 2017
*Enterococcus* sp.	Yellow tail (*Seriola quinqueradiata*)	Kusuda and Salati, 1993
	Turbot (*Scophthalmus maximus*)	Nieto et al., 1995
	Nile tilapia (*Oreochromis niloticus*)	Plumb and Hanson, 2010
*L. garvieae*	Red sea wrasse (*Coris aygula*)	Colorni et al., 2003
	Nile tilapia and Pintado (*Pseudoplathystoma corruscans*)	Evans et al., 2009
	Rainbow trout (*Oncorhynchus mykiss*)	Aguado-Urda et al., 2011; Reimundo et al., 2011
	Gray mullet (*Mugil cephalus*)	Chen et al., 2002
	Catfish (*Silurus glanis*)	Ravelo et al., 2003
	Freshwater prawn (*Macrobrachium rosenbergii*)	Shih-Chu et al., 2001
*Carnobacterium* sp.	Rainbow trout Striped bass and channel catfish Salmon Lake white fish	Hiu et al., 1984; Baya et al., 1991; Starliper et al., 1992; Toranzo et al., 1993b Baya et al., 1991; Toranzo et al., 1993a Michel et al., 1986 Loch et al., 2008

**Table 6:** Treatments for pathogenic LAB in aquaculture.

**LAB species/treatments**	**Type of treatments**	**Studied species**	**References**
***Streptococcus agalactiae***			
Vaccine	Feed-based recombinant	Tilapia (*Oreochromis* sp.)	Nur-Nazifah et al., 2014
	Oral DNA	Nile tilapia (*Oreochromis niloticus*)	Huang L. Y. et al., 2014; Ma et al., 2017; Zhu et al., 2017
	FbsA and α-enolase	Nile tilapia	Yi et al., 2014
	SAΔ*phoB* live attenuated vaccine	Golden pompano (*Trachinotus ovatus*)	Cai et al., 2017
	PLGA-LrrG protein microparticle	Nile tilapia	Ke et al., 2017
	GapA protein	Nile tilapia	Zhang Z. et al., 2017
Medical herbs	*Sophora flavescens root extract*	Nile tilapia	Wu et al., 2013
	Liposome-encapsulated cinnamaldehyde	Zebrafish (*Danio rerio*)	Faikoh et al., 2014
	Essential oils	Nile tilapia	Brum et al., 2017
	*Excoecaria agallocha* leaf extracts	Nile tilapia	Laith et al., 2017
Probiotics	*B. subtilis*	Tilapia	Ng et al., 2014; Liu et al., 2017
	*B. pumilus*	Nile tilapia	Srisapoome and Areechon, 2017
	*Saccharomyces cerevisiae*	Nile tilapia	Pinpimai et al., 2015
	*Lb. rhamnosus*	Nile tilapia	Pirarat et al., 2015
Prebiotics	β-glucan	Nile tilapia	Pilarski et al., 2017
	*Cordyceps militaris* spent mushroom substrate	Nile tilapia	Van Doan et al., 2017a,b
Probiotics + Prebiotics	Kefir + low molecular weight sodium alginate	Nile tilapia	Van Doan et al., 2017b
	*Cordyceps militaris* spent mushroom substrate + *Lb. plantarum*	Nile tilapia	Van Doan et al., 2017a
***Streptococcus iniae***			
Vaccine	Formalin-killed cells Live *S. iniae* mutant strain *Lb. lactis* BFE920-SiMA feed vaccine DNA vaccines (pEno)	Nile tilapia Nile tilapia Olive flounder (*Paralichthys olivaceus*) Nile tilapia	Klesius et al., 2000 Wang et al., 2014 Kim et al., 2016 Kayansamruaj et al., 2017
Medical herbs	Inositol Essential oils Aloe vera *Spirulina platensis*		Peres et al., 2004 Soltani et al., 2014 Gabriel et al., 2015 Adel et al., 2016
Probiotics and prebiotics	Grobiotic™AE *B. subtilis* and *Lb. acidophilus*	Hybrid striped bass (*Morone chrysops x M. saxatilis*) Nile tilapia	Li and Gatlin, 2004 Aly et al., 2008
	*L. lactis*	Olive flounder	Kim et al., 2013
	*B. subtilis, S. cerevisiae and Aspergillus oryzae*	Nile tilapia	Iwashita et al., 2015
	*E. casseliflavus*	Rainbow trout (*Oncorhynchus mykiss*)	Safari et al., 2016
Nucleotides	Oligonucleotides	Hybrid striped bass	Li et al., 2004
	Nucleotides	Rainbow trout	Tahmasebi-Kohyani et al., 2011
Vitamins	Vitamin E	Nile tilapia	Lim et al., 2009
	Vitamin A	Nile tilapia	Guimarães et al., 2014
*S. dysgalactiae* *S. parauberis* *S. uberis*	Not available	Not available	Not available
***Enterococcus faecalis***			
*Medical plants*	*Tamarindus indica* and *Emblica officinalis* leaves, *Allium sativum* bulb, and *Syzygium aromaticum* bud extracts	Nile tilapia, freshwater catfish (*Clarias batrachus*) and Asian stinging catfish (*Heteropneustes fossilis*)	Rahman et al., 2017
***Lactococcus garvieae***			
Vaccine	Autogenous formalin-inactivated Inactivated vaccine Ichtiovac-Lg Bivalent vaccine Subunit vaccines	Tilapia and rainbow trout Rainbow trout Rainbow trout and Olive flounder	Bercovier et al., 1997 Vendrell et al., 2007 Bastardo et al., 2012 Ra et al., 2009
Medical herbs	Essential oils Mushroom extracts Stinging nettle Extract of noni leaves Huanglian Jiedu decoction	Rainbow trout Rainbow trout Rainbow trout Freshwater prawn (*Macrobrachium rosenbergii*) Gray mullet (*Mugil cephalus*)	Soltani et al., 2015 Baba et al., 2015 Saeidi Asl et al., 2017 Marisa Halim et al., 2017 Choi et al., 2014
Antibiotics	Lincomycin, tetracycline chloramphenicol Erythromycin, lincomycin, and oxytetracycline Erythromycin, oxytetracycline, and amoxicillin	Yellow tail (*Seriola quinqueradiata*) Yellow tail	Aoki et al., 1990 Kawanishi et al., 2005 Vendrell et al., 2006
*Carnobacterium* sp.	Not available	Not available	Not available

### streptococcus

*Streptococcus* spp. is the most common pathogen in aquaculture, and up to date, several species within this genus have been reported as important pathogens of fish, such as silver pomfret (*Pampus argenteus*) (Duremdez et al., 2004), red tilapia (*O. niloticus*) (Musa et al., 2009), golden pompano (*Trachinotus blochii*) (Amal et al., 2012), barcoo grunter (*Scortum barcoo)* (Liu et al., 2014), and hybrid tilapia (*O. niloticus* × *O. aureus)* (Al-Harbi, 2016).

Infection of *Streptococcus agalactiae* led to persistent high mortality with a distinctive swollen belly, eye hemorrhages, corneal opacity, exophthalmia, hemorrhage, enlarged liver and congestion of the kidney and spleen (Duremdez et al., 2004; Amal et al., 2012; Liu et al., 2014; Al-Harbi, 2016). To deal with this bacterial strain, several type of vaccines have been developed, which include formalin-killed cells and concentrated extracellular products of a single isolate of *S. agalactiae* vaccine (Evans et al., 2004), feed-based recombinant vaccine encoding cell wall surface anchor family protein of *S. agalactiae* (Nur-Nazifah et al., 2014), oral DNA vaccine (Huang L. Y. et al., 2014; Ma et al., 2017; Zhu et al., 2017), FbsA and α-enolase (Yi et al., 2014), SAΔ*phoB* live attenuated vaccine (Cai et al., 2017), PLGA-LrrG protein micro-particle vaccine (Ke et al., 2017), and GapA protein vaccine (Zhang Z. et al., 2017). In addition to vaccines, many functional feed additives have been proved to protect fish and shellfish against *S. agalactiae* such as Ku shen (*Sophora flavescens*) root extract (Wu et al., 2013), liposome-encapsulated cinnamaldehyde (Faikoh et al., 2014), *B. subtilis* and *B. pumilus* Ng et al., 2014; Liu H. et al., 2017; Srisapoome and Areechon, 2017, yeast (*Saccharomyces cerevisiae*) (Pinpimai et al., 2015), *Lb. rhamnosus* (Pirarat et al., 2015), essential oils (Brum et al., 2017), buta–buta (*Excoecaria agallocha*) leaf extracts (Laith et al., 2017), β-glucan (Pilarski et al., 2017), kefir, low molecular weight sodium alginate, and *Lb. plantarum* Van Doan et al., 2016a,b, 2017b, and scarlet caterpillar (*Cordyceps militaris*) spent mushroom substrate and *Lb. plantarum* (Doan et al., 2017; Van Doan et al., 2017a). *S. iniae* is another *Streptococcus* species that cause disease outbreaks in different fish species (Agnew and Barnes, 2007), hybrid striped bass (*Morone chrysops* × *Morone saxatilis)* (Stoffregen et al., 1996), rabbitfish (*Siganus canaliculatus)* (Yuasa et al., 1999), European sea bass (Colorni et al., 2002), Japanese flounder (Nguyen et al., 2002), barramundi (*L. calcarifer)* (Bromage et al., 1999), and hybrid tilapia (Al-Harbi, 2011). Infection of this bacterium has led to vast economic losses in the world aquaculture industry of ~150 million US$, annually (Shoemaker et al., 2001; Al-Harbi, 2011). Huge effort has been contributed to deal with this bacterium which include vaccine (Klesius et al., 2000; Wang et al., 2014; Kim et al., 2016; Kayansamruaj et al., 2017), probiotics (*B. subtilis, Lb. acidophilus, L. lactic, E. casseliflavus, S. cerevisiae, and Aspergillus oryzae*) and prebiotics (Grobiotic^TM^AE) (Li and Gatlin, 2004; Aly et al., 2008; Kim et al., 2013; Iwashita et al., 2015; Safari et al., 2016), medicinal plants (inositol, essential oil, Aloe vera, and *Spirulina platensis*) (Peres et al., 2004; Soltani et al., 2014; Gabriel et al., 2015; Adel et al., 2016), nucleotides (Li et al., 2004; Tahmasebi-Kohyani et al., 2011), and vitamins(A and E) (Lim et al., 2009; Guimarães et al., 2014). Besides these two common pathogens, several species within genus *Streptococcus* such as *Streptococcus dysgalactiae* (Yang and Li, 2009), *S. parauberis* (Haines et al., 2013), and *Streptococcus uberis* (Luo et al., 2017) have been reported to be pathogenic in aquaculture. However, to our knowledge, there is no treatment against these species.

### enterococcus

*Enterococcus* sp. is an important pathogen in aquaculture, with severely impacts in commercial aquaculture practices worldwide (Martins et al., 2008; Rahman et al., 2017). The first report on the occurrence of pathogenic *Enterococcus* sp. in fish was revealed in yellow tail (*Seriola quinqueradiata*) in Japan (Kusuda and Salati, 1993). Later, *Enterococcus* was revealed in turbot (*S. maximus*) (Nieto et al., 1995), and tilapia (*O. niloticus*) (Plumb and Hanson, 2010). *E. faecalis* has been reported as causative agent of streptococcal infection in tilapia in lakes of Egypt, Thailand, and Bangladesh (Petersen and Dalsgaard, 2003; Abou El-Geit et al., 2013; Rahman et al., 2017). To our knowledge, limited information regarding prevention and treatment methods against *E*. *faecalis* has been reported. However, recently, Rahman et al. (2017) demonstrated that extraction of some medicinal plants, such as tamarind (*Tamarinds indica*), Indian gooseberry (*Phyllanthus emblica*), garlic (*Allium sativum*), and clove (*Syzygium aromaticum*) significantly protected fish against *E*. *faecalis* infection.

### lactococcus garvieae

The pathogenicity of *L. garvieae* is well-known (Vendrell et al., 2006; Michel et al., 2007; Fukushima et al., 2017; Meyburgh et al., 2017) and the bacterium is the causative agent of lactococcosis, a hyperacute haemorrhagic septicaemia of fish. Huge economic loss in several economical freshwater - and marine fish species has been reported as a result of lactococcosis infection in Red sea wrasse (*Coris aygula*) (Colorni et al., 2003), Nile tilapia and pintado (*Pseudoplathystoma corruscans*) (Evans et al., 2009), rainbow trout (Aguado-Urda et al., 2011; Reimundo et al., 2011), gray mullet (*Mugil cephalus*) (Chen et al., 2002), catfish (*Silurus glanis*) (Ravelo et al., 2003), and freshwater prawn (*Macrobrachium rosenbergii*) (Shih-Chu et al., 2001). The common way to deal with this bacterium was the use of antibiotic, such as lincomycin, oxytetracycline and macrolides (Aoki et al., 1990; Kawanishi et al., 2005), and erythromycin, oxytetracycline, amoxicillin, and doxycycline have been widely used to control outbreaks of lactococcosis through rainbow trout (Vendrell et al., 2006). It is known that antibiotics were highly effective against *L. garvieae* in *in vitro* studies, but not in field conditions because of anorexia of infected fish (Bercovier et al., 1997) and possibly by ineffective metabolism of antibiotics in fish (Meyburgh et al., 2017). Due to this limitation of antibiotics and their side-effects in aquaculture practice, vaccination was considered as most effective to control lactococcosis (Meyburgh et al., 2017). Several types of vaccine have been developed such as autogenous formalin-inactivated vaccines (Bercovier et al., 1997), inactivated vaccine Ichtiovac-Lg (Vendrell et al., 2007), bivalent vaccine (Bastardo et al., 2012), and subunit vaccines (Ra et al., 2009). In addition to vaccines, several functional feed additives have been demonstrated to protect the fish against this bacterium which include essential oil (Soltani et al., 2015), mushroom extracts (Baba et al., 2015), stinging nettle (Saeidi Asl et al., 2017), extract of noni leaves (Marisa Halim et al., 2017), and Huanglian Jiedu decoction (Choi et al., 2014).

### carnobacterium

*C. maltaromaticum* was reported as an important species and is reported in numerous fish species and meat products (Leisner et al., 2012). This bacterium has been demonstrated as a promising probiotic for aquaculture (Ringø et al., 2005; Kim and Austin, 2008; Pikuta and Hoover, 2014). However, some strains of this bacterium has been reported as fish pathogens with low virulence and stressed fish are especially susceptible, particularly post spawning (Michel et al., 1986; Starliper et al., 1992). Several fish species has been infected with *C. maltaromaticum* such as rainbow trout (Hiu et al., 1984; Baya et al., 1991; Starliper et al., 1992; Toranzo et al., 1993b), striped bass and channel catfish (Baya et al., 1991; Toranzo et al., 1993a), salmon (Hiu et al., 1984; Michel et al., 1986), and lake whitefish (Loch et al., 2008). However, to our knowledge, there is no information available regarding prevention and treatment approaches of this bacterium in aquaculture.

## Practical uses of LAB as an immunostimulant in finfish aquaculture

Finfish share many common structures and functions with warm-blooded animals in innate immunity (Whyte, 2007), adaptive immunity (Laing and Hansen, 2011), and mucosal immunity (Gomez et al., 2013), although apparent differences exist. The finfish immune systems are regulated in the same or very similar manners to those of other vertebrates. Since antibiotics have significant limitations in finfish aquaculture, the field has sought safer and more effective antibiotic alternatives. Natually, LAB became a candidate for a substitute for antibiotics because the immunostimulant effects of LAB have been well established in other animals including human.

Various strains of LAB have been studied in their immune modulatory effects on many different finfish species; summarized in Tables 7, 8. Genus *Lactobacillus* is most studied (Salinas et al., 2006; Balcázar et al., 2007b; Picchietti et al., 2009; Harikrishnan et al., 2011; Biswas et al., 2013; Giri et al., 2013; Liu et al., 2013; Gioacchini et al., 2014; Van Doan et al., 2014, 2016a,b; Beck et al., 2015, 2016; Mohammadian et al., 2016; Lee et al., 2017; Zhang Z. et al., 2017). The second most investigated genus is *Lactococcus* (Balcázar et al., 2007b; Kim et al., 2013; Beck et al., 2015, 2016; Nguyen et al., 2017). Genera of *Enterococcus, Pediococcus*, and *Leuconostoc* have also been studied at a significant level; *Enterococcus* (Wang et al., 2008; Kim et al., 2012; Rodriguez-Estrada et al., 2013; Matsuura et al., 2017), *Pediococcus* (Neissi et al., 2013; Dawood et al., 2016a; Kaew-on et al., 2016), *Leuconostoc* (Balcázar et al., 2007b). Although the majority of the studies used a specific strain of live LAB (Table 7), some studies were performed with their inactivated form of LAB (Table 8). The immunostimulant effects of a mixture of two different LAB were also investigated. These studies revealed that the mixture LAB were superior to a single homogenous LAB in the probiotic effects (Beck et al., 2015; Maji et al., 2017). Not only various strains of LAB, but diverse species of subject fish were investigated as well; olive flounder, Nile tilapia, shirbot, Huanghe common carp (*C. carpio* Huanghe var.), European sea bass; basa fish (*P. bocourti*), Japanese eel (*Anguilla japonica*), rohu, zebrafish, striped beakfish (*Oplegnathus fasciatus*), rainbow trout, green terror (*A. rivulatus*), goldfish (*Carassius auratus*), gilthead sea bream, tiger puffer (*Takifugu rubripes*) and red sea bream.

**Table 7:** Immunological changes of finfish resulted by live LAB treatment.

**LAB**	**Fish model (weight)**	**Administration route and dose**	**Administration length**	**Immunological changes**	**References**
*E. faecium* (strain not mentioned)	Olive flounder (*Paralichthys olivaceus*) (33.4 ± 10 g)	Intraperitoneal injection 10^9^ CFU/fish	15 days	Alternative complement activity ↑, Serum lysozyme activity ↑, Spleen: *IL-1β* ↑, Kidney: *IL-1β* ↑, *TNF-α* ↑	Kim et al., 2012
*E. faecium* ZJ4	Nile tilapia (*Oreochromis niloticus*) (6.834 ± 0.18 g)	Immersion 10^7^ CFU/mL supplemented in aquaria for every 4 days	40 days	Complement C3 ↑, Myeloperoxidase activity ↑, NBT reaction (respiratory burst) ↑, Serum lysozyme activity ↑,	Wang et al., 2008
*Lb. acidophilus* JCM 1132	Nile tilapia (*Oreochromis niloticus* ♀ × *Oreochromis aureus ♂*) (0.9 g)	Diet 10^5^, 10^7^, 10^9^ CFU/g feed	10, 20, 35 days (consecutive)	Spleen: *IL-1β* ↑*, TGF-β* ↑,*TNF-α* ↑ at day 20, *TNF-α* ↓ at day 35 Kidney: *IL-1β* ↑ at day 20, *IL-1β* ↓ at day 35, *TGF-β* ↑,*TGF-β* ↓ at day 35 in 10^5^ CFU/g feed, *TNF-α* ↑ Protection against *A. hydrophila* ↑ * Increased or decreased gene expressions were varied by dose and sample collection time mark.	Liu et al., 2013
*Lb. brevis* JCM 1170	Nile tilapia (0.9 g)	Diet 10^5^, 10^7^, 10^9^ CFU/g feed	10, 20, 35 days (consecutive)	Spleen: *IL-1β* ↑, *TGF-β* ↑ at day 20, *TGF-β* ↓ at day 35, *TNF-α* ↑ at day 20, *TNF-α* ↓ at day 35 Kidney: *IL-1β* ↑ at day 20, *IL-1β* ↓ at day 35, *TGF-β* ↑ at day 20, *TNF-α* ↑ at day 35 Protection against *A. hydrophila* ↑ * Increased or decreased gene expressions were varied by dose and sample collection time mark.	Liu et al., 2013
*Lb. casei* PTCC1608	Shirbot (*Barbus grypus*) (50 g)	Diet 5 × 10^7^ CFU/g feed	6 weeks	Alternative complement activity ↑, NBT reaction (respiratory burst) ↑, Protection against *A. hydrophila* ↑	Mohammadian et al., 2016
*Lb. delbrueckii* (Angel Company, Wuhan, China)	Huanghe common carp (*Cyprinus carpio* Huanghe var.) (1.05 ± 0.03 g)	Diet 10^5^, 10^6^, 10^7^, 10^8^ CFU/g feed	8 weeks	Intestine: *IL-1β* ↓, IL-8 ↓, *TNF-α* ↓, *NF-κB P65* ↓, *IL-10* ↑, *TGF-β* ↑ IgM concentration ↑, Myeloperoxidase activity ↑, Serum lysozyme activity ↑, Protection against *A. hydrophila* ↑	Zhang C.-N. et al., 2017
*Lb. delbrueckii* ssp. *delbrueckii* AS13B	European sea bass (*Dicentrarchus labrax* (L.)) (not available, 1 day post hatch)	Diet 10^5^ CFU/cm^3^ via enriched in *Brachionus plicatilis* or *Artemia nauplii*	72 days	Acidophilic granulocytes ↑, T cells ↑, *IL-1 β* ↓	Picchietti et al., 2009
*Lb. delbrueckii* ssp*. bulgaricus* (original isolate by authors)	Shirbot (50 g)	Diet 5 × 10^7^ CFU/g feed	6 weeks	Alternative complement activity ↑, NBT reaction (respiratory burst) ↑, Protection against *A. hydrophila* ↑	Mohammadian et al., 2016
*Lb. plantarum* (original isolate by authors)	Shirbot (50 g)	Diet 5 × 10^7^ CFU/g feed	6 weeks	Alternative complement activity ↑, NBT reaction (respiratory burst) ↑, Serum lysozyme activity (only at day 60) ↑, Protection against *A. hydrophila* ↑	Mohammadian et al., 2016
*Lb. plantarum* CR1T5	Basa fish (*Pangasius bocourti*) (82.01 g)	Diet 10^8^ CFU/g feed	4 weeks	Alternative complement activity ↑, Protection against *A. hydrophila* ↑	Van Doan et al., 2014
*Lb. plantarum* CR1T5	Basa fish (3.57 g)	Diet 10^8^ CFU/g feed	3, 6, 9, 12 weeks (consecutive)	Alternative complement activity ↑, Phagocytic activity ↑, Serum lysozyme activity ↑, Protection against *A. hydrophila* ↑	Van Doan et al., 2016a
*Lb. plantarum* CR1T5	Nile tilapia (15.56 ± 0.02 g)	Diet 10^8^ CFU/g feed	30 and 60 days (consecutive)	Alternative complement activity ↑, NBT reaction (respiratory burst) ↑, Phagocytic activity ↑, Serum lysozyme activity ↑, Protection against *S. agalactiae* ↑	Van Doan et al., 2016b
*Lb. plantarum* FGL0001	Olive flounder (37.5 ± 1.26 g)	Diet 10^7^ CFU/g feed	4 weeks	NBT reaction (respiratory burst) ↑, Phagocytic activity ↑, Skin mucus lysozyme activity ↑, Intestine: *IL-6* ↑, *IL-8* ↑, *TNF-α* ↑, Protection from *S. iniae* ↑	Beck et al., 2015
*Lb. plantarum* FGL0001	Olive flounder (42.7 ± 1.61 g)	Diet 10^7^ CFU/g feed	4 weeks	Intestine: CD4-1 ↑, T-bet ↑, GATA3 ↑, IL-1β ↑, IFN-γ ↑, IL-17A/F ↓, Gut permeability ↓, Protection from *E. tarda* ↑	Beck et al., 2016
*Lb. plantarum* KCTC3928	Japanese eel (*Anguilla japonica*) (8.29 ± 00.6 g)	Diet 10^6^, 10^7^, 10^8^ CFU/g feed	8 weeks	Myeloperoxidase activity (10^8^ CFU/g feed only) ↑, Serum lysozyme activity ↑, Superoxide dismutase ↑, Intestine: *IgM* ↑, Protection from *V. anguilarum* (10^8^ CFU/g feed only) ↑	Lee et al., 2017
*Lb. plantarum* VSG3	Rohu (60 g)	Diet 10^6^, 10^8^, 10^10^ CFU/g feed	30 and 60 days (consecutive)	Alternative complement activity ↑, IgM concentration at 30^th^ day (10^8^ and 10^10^ CFU/g feed) ↑ NBT reaction (respiratory burst) ↑, Phagocytic activity ↑, Serum lysozyme activity ↑, Protection from *A. hydrophila* ↑	Giri et al., 2013
*Lb. rhamnosus* IMC 501	Zebrafish (*Danio rerio*) (adult, weight is not mentioned)	Diet 10^6^ CFU/g feed	10 days	Liver: *IL-1β* ↑, *TNF-α* ↑	Gioacchini et al., 2014
*Lb. sakei* BK19	Striped beakfish (*Oplegnathus fasciatus*) (32 ± 3 g)	Diet 2.2 × 10^7^ CFU/g feed	1, 3, 6 weeks (consecutive)	Alternative complement activity ↑, Eosinophils ↑, Monocytes ↑, NBT reaction (respiratory burst) ↑, Neutrophils ↑, Reactive nitrogen species ↑, Serum lysozyme activity ↑	Harikrishnan et al., 2011
*Lb. sakei* CLFP 202	Rainbow trout (40 g)	Diet 10^6^ CFU/g feed	2 weeks	Alternative complement activity ↑, Phagocytic activity ↑, Serum lysozyme activity ↑, Protection from *A. salmonicida* ↑	Balcázar et al., 2007b
*L. lactis* BFE920	Olive flounder (37.5 ± 1.26 g, 40 ± 3 g, 55 ± 5 g)	Diet 10^7^ CFU/g feed	4 weeks	Myeloperoxidase activity ↑, NBT reaction (respiratory burst) ↑, Phagocytic activity ↑, Skin mucus lysozyme activity ↑, Spleen: IL-12p40 ↑, IFN-γ ↑, Intestine: IL-6 ↑, IL-8 ↑, Protection from *S. iniae* ↑	Kim et al., 2013; Beck et al., 2015
*L. lactis* BFE920	Olive flounder (42.7 ± 1.61 g)	Diet 10^7^ CFU/g feed	4 weeks	Intestine: CD4-1 ↑, FOXP3 ↑, IL-10 ↑, TGF-β1 ↑, IFN-γ ↑, RORγ ↓, IL-17A/F ↓, Gut permeability ↓, Protection from *E. tarda* ↑	Beck et al., 2016
*L. lactis* ssp. *lactis* CLFP 100	Rainbow trout (40 g)	Diet 10^6^ CFU/g feed	2 weeks	Alternative complement activity ↑, NBT reaction (respiratory burst) ↑, Phagocytic activity ↑, Serum lysozyme activity ↑, Protection from *A. salmonicida* ↑	Balcázar et al., 2007b
*L. lactis* WFLU12	Olive flounder (80.84 ± 9.37 g)	Diet 10^9^ CFU/g feed	2, 4, 8 weeks (consecutive)	Intestine: *IL-6* (at week 4) ↑ Kidney: IL-6 (at week 2) ↑, IL-8 (at week 4) ↑, IFN-γ (at week 4) ↑, g-lysozyme (at week 4) ↑ Phagocytic activity (at week 2) ↑, NBT reaction (respiratory burst, at week 4) ↑, Natural infection of *S. parauberis* ↓	Nguyen et al., 2017
*Lc. mesenteroides* CLFP 196	Rainbow trout (40 g)	Diet 10^6^ CFU/g feed	2 weeks	Alternative complement activity ↑, Phagocytic activity ↑, NBT reaction (respiratory burst) ↑, Serum lysozyme activity ↑, Protection from *A. salmonicida* ↑	Balcázar et al., 2007b
*P. acidilactici* MA 18/5 M	Green terror (*Aequidens rivulatus*) (0.388 ± 0.0021 g)	Diet 0.9 × 10^7^ CFU/g feed	56 days	Alternative complement activity ↑, Serum lysozyme activity ↑, Total immunoglobulin counts ↑	Neissi et al., 2013
*P. pentosaceus* PKWA-1	Nile tilapia (0.68 ± 0.02 g, 36.89 ± 3.34 g)	Diet 10^7^ CFU/g feed	1, 14, 28, 42 days (consecutive)	Alternative complement activity ↑, Phagocytic activity ↑, Serum lysozyme activity ↑, Total leukocyte counts ↑, Protection from *A. hydrophila* ↑	Kaew-on et al., 2016
Mixed LAB (*Lb. plantarum* FGL0001, *L. lactis* BFE920)	Olive flounder (37.5 ± 1.26 g)	Diet 10^7^ CFU/g feed of each strain	4 weeks	NBT reaction (respiratory burst) ↑, Phagocytic activity ↑, Skin mucus lysozyme activity ↑, Intestine: IL-6 ↑, IL-8 ↑, TNF-α ↑, Protection from *S. iniae* ↑	Beck et al., 2015
Mixed LAB (*Lb. plantarum* SM16, *Lb. plantarum* SM33, *Lb. fermentum* SM51, *Lb. brevis* SM56, *P. pentosaceus* SM65)	Rohu (19.72 ± 0.18 g)	Diet 2 × 10^8^ CFU/g feed of each strain	30 days	NBT reaction (respiratory burst) ↑, Intestine and liver: TNF-α ↑, IL-10 ↑ Protection from *A. hydrophila* ↑	Maji et al., 2017

**Table 8:** Immunological changes of finfish resulted by inactivated LAB treatment.

**LAB**	**Fish model (weight)**	**Administration route and dose**	**Administration length**	**Immunological changes**	**References**
*E. faecalis* (Nichinichi Pharmaceutical, Japan)	Rainbow trout (*Oncorhynchus mykiss*) (36.3 ± 0.42 g)	Diet 0.25, 0.5% w/w inclusion to feeds	12 weeks	Mucus secretion ↑, Phagocytic activity ↑, Protection from *A. salmonicida* ↑ Systemic invasion of *A. salmonicida* ↓	Rodriguez-Estrada et al., 2013
*E. faecalis* KH2	Goldfish (*Carassius auratus*)(15–20 g) *In vitro*, kidney leukocytes	*In vivo*, intraperitoneal injection 500 μg/fish *In vitro* treatment 50 μg/well	*In vivo*, 7 days; *In vitro*, 12 h	In vivo: CD4-1^+^ cells ↑, CD8α^+^ cells ↑, Myeloid cells ↑, Macrophages ↑, *IL-12p35* ↑, IL-12p40 ↑, IFN-γ1 ↑ *In vitro*: IL4/13a ↑, IL-12p35 ↑, IL-12p40 ↑, IFN-γ1 ↑, IFN-γ2 ↑, infgrel1 ↑, infgrel2 ↑	Matsuura et al., 2017
*Lb. delbrueckii* ssp*. lactis* CECT287	Gilthead sea bream (*Sparus aurata* L.)(65 g) *In vitro*, head kidney cells	*In vitro* treatment 5 × 10^5^, 5 × 10^6^ 5 × 10^7^ CFU/mL	30 min	Respiratory burst ↑, Natural cytotoxic activity ↑,	Salinas et al., 2006
*Lb. paracasei* spp. *paracasei* 06TCa22	Tiger puffer (*Takifugu rubripes*)(205 ± 8 g) *In vitro*, head kidney cells	*In vitro* treatment 20 μg/mL	1, 4, 8, 12, 24, 48 h	IL-1β ↑, IL-2 ↑, IL-6 ↑, IL-7 ↑, IL-12p40 ↑, IL-17AF-3 ↑, IL-18 ↑, TNF-α ↑, TNF-N ↑, I-IFN-1 ↑, IFN-γ ↑	Biswas et al., 2013
*Lb. plantarum* 06CC2	Tiger puffer (205 ± 8 g) *In vitro*, head kidney cells	*In vitro* treatment 20 μg/mL	1, 4, 8, 12, 24, 48 h	IL-1β ↑, IL-2 ↑, IL-6 ↑, IL-7 ↑, IL-12p40 ↑, IL-10 ↑, IL-15 ↑, IL-18 ↑, IL-21 ↑, TNF-α ↑, TNF-N ↑, I-IFN-1 ↑	Biswas et al., 2013
*P. pentosaceus* D3268	Red sea bream (*Pagrus major*) (6 ± 0.2 g)	Diet 1.6 × 10^10^, 1.6 × 10^11^, 1.6 × 10^12^, 3.2 × 10^12^ CFU/g feed	56 days	Mucus lysozyme activity ↑, Mucus secretion ↑, Serum lysozyme activity ↑	Dawood et al., 2016a

The mode of administration of LAB is an important factor for practical use of LAB in the field. Feeding the LAB adsorbed into regular diets may be the best way for administration because this feeding method reduces labor and stress to fish. As expected, a vast majority of studies employed dietary LAB as the mode of administration. However, some studies treated the fish by intraperitoneal injection (Kim et al., 2012; Matsuura et al., 2017) or immersion in a LAB-containing bath (Wang et al., 2008). Many studies indicated that the feeding administration demonstrated better immunostimulant effects, compared to any other modes of application. The viability of LAB is another important issue to consider. The viability of microbes is a necessity for probiotics by definition. In general, live LAB triggered higher immune stimulation compared to that of the inactivated LAB (Panigrahi et al., 2005; Munoz-Atienza et al., 2015; Tables 7, 8). However, more studies that compare the activities between the live and the inactivated condition of the same LAB need to be done for further confirmation. Nevertheless, only live LAB can produce bioactive products such as exopolysaccharides and maintain the natural state of microbe-associated molecular patterns (MAMP) structures. These unique properties of live LAB may contribute to the superiority in immunostimulant effects over the inactivated form of the LAB. In this context, the establishment of proper techniques for storing and applying live LAB is an important aspect to consider. As summarized in Table 8, the inactive LAB also showed significant immunostimulant effects, but less than those of live LAB. However, in the aspect of manufacturing LAB products, the inactivated condition of LAB may be advantageous because the cost for storage and distribution can be reduced. For the practical utilization of LAB in the finfish aquaculture field, the species, the living status, the mode of administration, and the optimum dosage of the LAB should be carefully considered for the best results.

## LAB effects on innate immunity

Innate immunity takes the place of the first line of defense toward a wide range of pathogens. The interaction between MAMP in microbes and pattern-recognition receptors (PRR) on innate immune cells is one of the critical initiators for activation of the innate immune system. Some probiotics that have immunostimulant activity such as LAB can protect the host from various pathogens by stimulating the immune system. The LAB studies of warm-blooded animals seem to influence the similar studies in finfish. However, the finfish studies were heavily biased toward the LAB effects on innate immunity as shown Tables 7, 8. Furthermore, most of the studies simply described the physiological status without exploring the specific immune subsets responsible for disease resistance or the underlying mechanism. The studies of the adaptive immune system are even more limited. Antibody was the only subject studied, and the studies concerning T cell responses were very few, if any. Understanding the regulatory mechanism of the finfish immune system is a big challenge to the field of finfish immunology. This understanding is essential for developing safe and potent immunological means for the protection and cure of fish diseases.

## Immune parameters for studying finfish immunity

The immune parameters that have frequently been used for studying finfish immunity are listed and briefly explained in Table 9. The innate immune parameters include complement activity, lysozyme, phagocytosis, and respiratory burst. The level of antigen-specific antibodies is mostly used for representing adaptive immune responses. The types and the levels of cytokines are important indicators of both innate and adaptive immune status of fish.

**Table 9:** Frequently measured immune parameters in finfish studies.

**Immune parameters**	**Functions**	**References**
Antibody	Produced by B cells Recognizes and binds to specific antigens of pathogens Neutralization of pathogens Opsonization of antibody bound pathogens Activation of the complement system Activation of antibody-dependent cellular cytotoxicity	Abbas et al., 2014
Cytokine	Signal proteins of host cells Activation of inflammation through proinflammatory cytokines (eg., IL-1β, INF-γ, TNF-α) Regulation of immune activities/anti-inflammation through regulatory cytokines (eg., IL-10, TGF-β)	Wang and Secombes, 2013; Abbas et al., 2014; Turner et al., 2014
Complement activity	Non-cellular immune response which is activated by antigen-specific antibodies or lectin Formation of membrane attack complexes (MAC) of the surface of pathogens Induction of inflammation at local infection sites Opsonization of pathogens by antibody binding or complement subunits to the surface of pathogens	Alexander and Ingram, 1992; Abbas et al., 2014
Lysozyme	Non-cellular immune response toward bacterial pathogens Hydrolysis β-(1, 4) glycosidic linkages in N-acetylmuramic acid and N-acetylglucosamine of bacterial cell wall peptidoglycan	Alexander and Ingram, 1992
Phagocytosis	Engulfing activity of phagocytic cells such as dendritic cells, macrophages, and monocytes Direct killing of pathogen by intracellular lysosome of phagocytic cells Antigen presentation of phagocytosed antigens to T cell by dendritic cells and macrophages	Abbas et al., 2014
Respiratory burst	Oxidative potential of innate cells Pathogen killing effect by reactive oxygen species including hydrogen peroxide, superoxide anions, and hydroxyl radicals	Abbas et al., 2014

## Cytokines as important immune modulators of finfish immunity

Cytokines are small proteins (~5–20 KDa) that are important in cell signaling. They act through receptors and are particularly important in the immune system because cytokines modulate the balance between humoral and cellular immune responses. Cytokines regulate the maturation, growth, and responsiveness of particular cell populations (Abbas et al., 2014; Turner et al., 2014). Many studies demonstrated the cytokine induction effects of LAB in various finfish models (Picchietti et al., 2009; Kim et al., 2012, 2013; Biswas et al., 2013; Liu et al., 2013; Beck et al., 2015, 2016; Matsuura et al., 2017; Nguyen et al., 2017; Zhang Z. et al., 2017). The cytokine profiles modified by LAB administration are summarized in Tables 7, 8. Increased expression of proinflammatory cytokines (e.g., IL-1β, IL-6, IL-8, or TNF-α) directly correlates to disease protection against challenged pathogens. This protective activity of inflammatory cytokines may be because of the potentiation of the host immune system, resulting in rapid and efficient responses to the invading pathogens (Wang and Secombes, 2013; Turner et al., 2014). However, excessive inflammation can cause acute inflammatory symptoms leading to the death of the host. Therefore, maintaining a balanced inflammation status is critical. IL-10, an anti-inflammatory cytokine, is a well-known immune regulator. Some strains of dietary LAB induced *IL-10* expression in finfish; Biswas et al. (2013) (*Lb. plantarum* 06CC2 treated *T. rubripes*), Beck et al. (2016) (*L. lactis* BFE920 treated *P. olivaceus*), and Maji et al. (2017) (a mixture of *Lb. plantarum* SM16, *Lb. plantarum* SM33, *Lb. fermentum* SM51, *Lb. brevis* SM56, *P. pentosaceus* SM65 treated *Labeo rohita*). Beck and co-authors demonstrated that LAB plays an important role in the establishment of the “immune tone” in the finfish gut. The immune tone is a higher status of immunological-readiness to combat against pathogens. LAB established the proinflammatory or anti-inflammatory immune tone in a strain-specific manner. The finfish in proinflammatory immune tone was able to protect the challenged pathogen better compared to those with an anti-inflammatory immune tone. However, the fish in anti-inflammatory immune tone gained more weight (Beck et al., 2016). Therefore, monitoring the types of cytokines expressed after LAB treatment may be important to maximize the beneficial effects of the LAB. The underlying mechanisms involved in the establishment of the two different types of immune tones and their relationships to the adaptive immune system need to be further investigated.

## LAB effects on adaptive immunity

In addition to innate immunity, LAB treatment also influenced the adaptive immunity of finfish. The fish fed with LAB increased total T cell numbers (Picchietti et al., 2009). The LAB also activated the subtype-specific factors of CD4^+^ T helper cells (Th1, Th2, Th17, and Treg cell) (Beck et al., 2016) and CD8^+^ cytotoxic T cells (Beck et al., 2016; Matsuura et al., 2017). The modification of T cell composition may be due to the cytokines released from various subsets of immune cells that are induced by the treated LAB. IL-12, IL-18, and IFN-γ act on Th1 cell differentiation and activation. IL-4, IL-13, IL-5 are involved in Th2 cells, and IL-17, IL-22, IL-21 promote Th17 cell differentiation. Treg cell differentiation is controlled by IL-10 and TGF-β (Abbas et al., 2014). The relationships between cytokines and immune cells are mutually regulated; cytokines secreted from stimulated immune cells control the same or other immune cells through signaling pathways. The responding immune cells then release cytokines accordingly (Knosp and Johnston, 2012). The cytokine networks are closely linked between the innate and the adaptive immune system as well. IL-10 released from activated M2 macrophages (Martinez and Gordon, 2014) influences Treg cell differentiation. Also, IL-12 released by activated DCs and macrophages stimulate Th1 cells and NK cells to release IFN-γ. This IFN-γ then activates DCs and macrophages. The LAB's roles involved in this kind of immune modulation have been well-demonstrated in warm-blooded animals (Delcenserie et al., 2008; Bron et al., 2012). Although it appears that LAB play similar roles in the finfish immune system, further studies are required.

## Conclusions

Numerous reports exist in finfish regarding the microbiota modulating effects of dietary modifications and the presence of LAB in the GI tract. However, when investigating the GI tract microbiota, one major concern occur; most studies evaluating the fish gut microbiota have focus to characterize the communities in the GI lumen (the allochthonous microbiota), while those bacteria that adhere to the mucosal surface (the autochthonous microbiota); which may be important in specialized physiological functions, remain uncharacterized. We therefore recommend more focus on the autochthonous gut microbiota in future studies.

Previous studies were based on culture-based approaches, but this may be question. Although there is a discussion over the value and need of using culture-based techniques vs. culture-independent approaches, it is apparent that viable cells are valuable to culture collections, in vaccine production, and as probiotics and synbiotics. During the last decades, 16S rRNA gene fingerprinting methods such as denaturing gradient gel electrophoresis (DGGE) have been widely used, but the DGGE method only detect 1–2% of the microbial diversity. Next-Generation Sequencing (NGS) has been used in recent years to examine the gut microbiome of humans, terrestrial and marine vertebrate including some finfish species. However, as NGS has only been used in few finfish species such as rainbow trout, Atlantic salmon, Siberian sturgeon, zebrafish and gilthead sea bream, we recommend that this technique is used to explore the gut bacterial community of finfish.

LAB and their bacteriocins are alternatives to chemicals and antibiotics as antimicrobial activities toward pathogens have been revealed. In some cases LAB and their bacteriocins may be used in combination with low dosages of antibiotics. As novel applications of LAB and bacteriocins are increasing; within prospects of anti-quorum sensing strategies and site-specific drug delivery, this topic merits further investigations.

As the specific bio-active compounds and mechanism behind the antagonism of LAB bacteriocins have rarely investigated, this merits further investigations to validate the health claims. Furthermore, as there may be risk of possible horizontal transfer of antibiotic resistance genes through LAB, the use of promising LAB must follow strict guidelines in addition to antimicrobial actions. As the efficacy of the bacteriocins is dictated by environmental factors, there is also a need to determine the effective conditions for application of each LAB bacteriocin (Balciunas et al., 2013).

Recent studies regarding probiotic administration as revealed beneficial effects on growth performance, immune responses and disease resistance. However, still there is limited information available about the exact mode of action on physiology of host organism. Although, there are some assumptions and speculations, this should be clarified in future through in depth studies. Also, different studies revealed varied results on different species. Considering the species-specific effects, there should be studies to determined optimum probiotic and inclusion level for each cultured species. During the recent years, there has been increased attention toward probiotics effects on mucosal parameters and expression of immune, and antioxidant related genes expression. The possible mode of action on gene expression profile merit further researches.

In addition to the numerous beneficial LAB, there are several pathogenic species within genera *Streptococcus, Enterococcus, Lactobacillus, Carnobacterium*, and *Lactococcus*. They have caused considerable losses in aquaculture practice. Huge effort been contributed to deal with these pathogens such as vaccines, dietary supplements; medicinal plants, prebiotics, probiotics and other immunostimulants. Such treatments needs to be developed in the future for sustainable aquaculture.

It is quite clear that LAB administration results in beneficial effects such as disease resistance and weight gain in finfish aquaculture. However, the underlying mechanism is poorly understood; the microbe-associated molecular patterns (MAMPs) of the LAB, their pattern recognition receptors (PRRs) on immune cells, and byproducts released from the LAB that are responsible for immunomodulation. The immunomodulatory effects of the LAB are strain-specific, and therefore, the information of the studies performed with various strains of LAB need to be further accumulated and actively shared for finfish aquaculture industries.

## Author contributions

ER: introduction, GI tract, editorial. KG: antibacterial effects of LAB. SH: LAB as probiotic. HD: pathogenic LAB. BB and SS: immunology of LAB.

### Conflict of interest statement

The authors declare that the research was conducted in the absence of any commercial or financial relationships that could be construed as a potential conflict of interest.
